# Three-Dimensional Simulation of Transient Resin Non-Isothermal Flow Across a Porous Reinforcement with a Sink Term: Capillary Number Evaluation in RTM Process

**DOI:** 10.3390/ma19173739

**Published:** 2026-09-02

**Authors:** João V. N. Sousa, João M. P. Q. Delgado, Adjalmir A. Rocha, Hortência L. F. Magalhães, Priscila S. Souza, Mylena M. D. O. Silva, Romário D. Moura, Fernando F. Vieira, Márcia R. Luiz, Ivonete B. Santos, Pablícia O. Galdino, Fernanda V. Amorim, Antonio G. B. Lima

**Affiliations:** 1 Postgraduate Program in Process Engineering, Federal University of Campina Grande, Campina Grande 58429-900, Brazil; joao.vns@hotmail.com; 2 CONSTRUCT, Department of Civil Engineering, Faculty of Engineering, University of Porto, 4200-465 Porto, Portugal; 3 Department of Civil Engineering, Federal University of Campina Grande, Campina Grande 58429-140, Brazil; adjalmir.arquiteto@gmail.com; 4 Science and Technology Institute, Federal University of the Vales do Jequitinhonha e Mucuri, Diamantina 39100-000, Brazil; hortencia.luma@ufvjm.edu.br; 5 Fundamental Chemistry Department, Institute of Chemistry, University of São Paulo, São Paulo 05508-000, Brazil; priscila.santos@iq.usp.br; 6 Department of Sanitary and Environmental Engineering, State University of Paraiba, Campina Grande 58429-500, Brazil; mylenamarilia@gmail.com (M.M.D.O.S.); romariodmoura@gmail.com (R.D.M.); fernando.desa@servidor.uepb.edu.br (F.F.V.); marciarluiz@servidor.uepb.edu.br (M.R.L.); 7 Department of Physics, State University of Paraiba, Campina Grande 58429-500, Brazil; ivoneetebs@gmail.com; 8 Department of Chemistry, State University of Paraiba, Campina Grande 58429-500, Brazil; pabliciagaldino@servidor.uepb.edu.br; 9 Department of Chemistry Teaching, Federal University of Pernambuco, Caruaru 55014-900, Brazil; fernanda.vieiraamorim@ufpe.br; 10 Department of Mechanical Engineering, Federal University of Campina Grande, Campina Grande 58429-900, Brazil; antonio.gilson@ufcg.edu.br

**Keywords:** non-isothermal RTM, void formation, polymer composite, CFD, fluid absorption, capillary number

## Abstract

Resin Transfer Molding (RTM) is a broadly applied composite fabrication technique in which liquid resin is injected into a sealed mold housing a fibrous preform. This technique enables the industrial-scale production of large, complex-shaped components while ensuring high quality and excellent material properties. A notable phenomenon in the Resin Transfer Molding process, particularly when vegetable fibers are used as reinforcement, involves resin absorption by the fibers during the filling process, which causes a deceleration of the fluid flow front, leading to an increase in mold filling time. This phenomenon has been scarcely investigated in the scientific literature, particularly under non-isothermal situations. In this context, this paper focuses on the resin injection in a closed and heated mold, emphasizing the evaluation of the capillary number of the fluid flow. The model incorporates a transient, radial, and 3D flow, variable resin viscosity, and includes the effect of resin absorption by the fibers. Simulations were carried out using CFD techniques, evaluating the effect of fixed injection gauge pressure (2.0 bar) or volumetric flow rate (3.5 × 10^−8^ m^3^/s) on the infiltration transient behavior. The numerical results of the capillary number of the flow, resin viscosity, and resin superficial velocity as a function of radial position were analyzed. Based on the analysis of the capillary number field, with the sole objective of minimizing the potential formation of voids in the manufactured composite, optimal operating parameters were identified, namely, a mold heating temperature of 30 °C and an optimal resin injection condition consisting of a constant volumetric flow rate of 3.5 × 10^−8^ m^3^/s. Further, the volumetric fraction, pressure, and temperature fields are presented and examined. The numerical modeling used builds upon a previously validated numerical framework and extends its application by evaluating the potential generation of voids through the analysis of the capillary number field.

## 1. Introduction

Modern technological demands often require materials with unique property combinations that cannot typically be achieved by metals, ceramics, or polymers alone. These requirements are effectively met through composite materials, which have found extensive use across diverse areas such as defense, automotive, aerospace, medical, sports, and consumer markets [1]. In aerospace, for instance, composites are applied in space stations, satellites, launch vehicles, robotic arms, spaceports, and cargo bay doors. Within the automotive sector, they are widely applied in brake systems, bumpers, clutch discs, rocker covers, and structural frames, among other applications. This trend is clearly demonstrated by the increasing use of composites over recent decades to substitute conventional materials, particularly in the aerospace sector [2]. In fact, the global composites market was valued at USD 106 billion in 2022 and is projected to grow to USD 191 billion by 2032 [3], underscoring both the current relevance and future significance of this industrial sector.

Among the various manufacturing methods for polymer matrix composites, liquid molding in closed molds stands out due to its industrial relevance, as it allows mass production with a lower environmental footprint than alternative methods [4]. Within this category, one of the most widely recognized processes is Resin Transfer Molding (RTM). This method involves injecting a low-viscosity thermoset resin into a closed mold that contains dry reinforcement fibers (preform) [5]. Polymer composite manufacturing using the RTM process has gained wide adoption due to its numerous advantages over other manufacturing methods [6,7]. Among these benefits are: reduced labor costs and production time; production of parts with high surface quality; compatibility with a wide variety of reinforcements; suitability for manufacturing parts with complex geometries; good dimensional tolerances in the final product; reduced environmental impact; and minimized risk of air entrapment within the product.

Several factors influence the RTM process, including mold shape, resin properties, fiber volume fraction, fiber–resin interactions, resin injection volumetric flow rate, injection pressure, air outlet locations, resin injection points, and mold temperature [8,9]. Careful control of these parameters is essential to ensure the production of high-quality composites while also reducing manufacturing costs and enhancing the final product’s performance.

As a result of the thermodynamics associated with the RTM process, resin viscosity changes as the injection progresses, representing a critical aspect of production control. Due to resin curing, there is an increase in its viscosity during infiltration, causing more difficult resin flow through the reinforcement, which can lead to incomplete filling of certain mold regions and an increase in the void fraction [8]. Consequently, this affects the mechanical performance of composite material. Heated molds lower the resin viscosity, enhancing the impregnation of the reinforcement and reducing the overall mold filling time [10].

Additionally, resin absorption by the reinforcement, namely, the resin infiltration into the micropores between individual fibers inside the fiber bundles, is a significant phenomenon in the RTM process [8,11]. A thorough comprehension and accurate representation of this absorption behavior are essential for advancing the RTM process, as they help minimize the void content in the final composite. This knowledge is of great relevance to both academia and industry, particularly in the context of producing high-quality composites required for demanding applications such as aeronautics and aerospace [12,13].

Of the numerous experimental studies on the RTM process [14,15,16,17,18,19,20], only a limited number specifically address the phenomenon of resin entrapment within the fiber reinforcements [21]. These studies have shown that the displacement of the resin in the fibrous reinforcement is affected by different physical parameters, such as resin properties and fiber architecture. The grouping of some of these parameters results in a dimensionless number known as the Capillary number, which expresses the relationship between viscous and capillary forces acting on the flow. There are several equations for determining the Capillary number, the most commonly used being the one described as follows [22]:

(1) $$Ca=\frac{\mu U}{\gamma }$$

where *Ca* represents the capillary number; *µ* is the resin viscosity at the flow front; *U* is the resin superficial velocity, measured at the flow front; in this work, measured at the position where the resin volume fraction is at least 1%; and *γ* is the surface tension of the resin.

**Figure 1:**
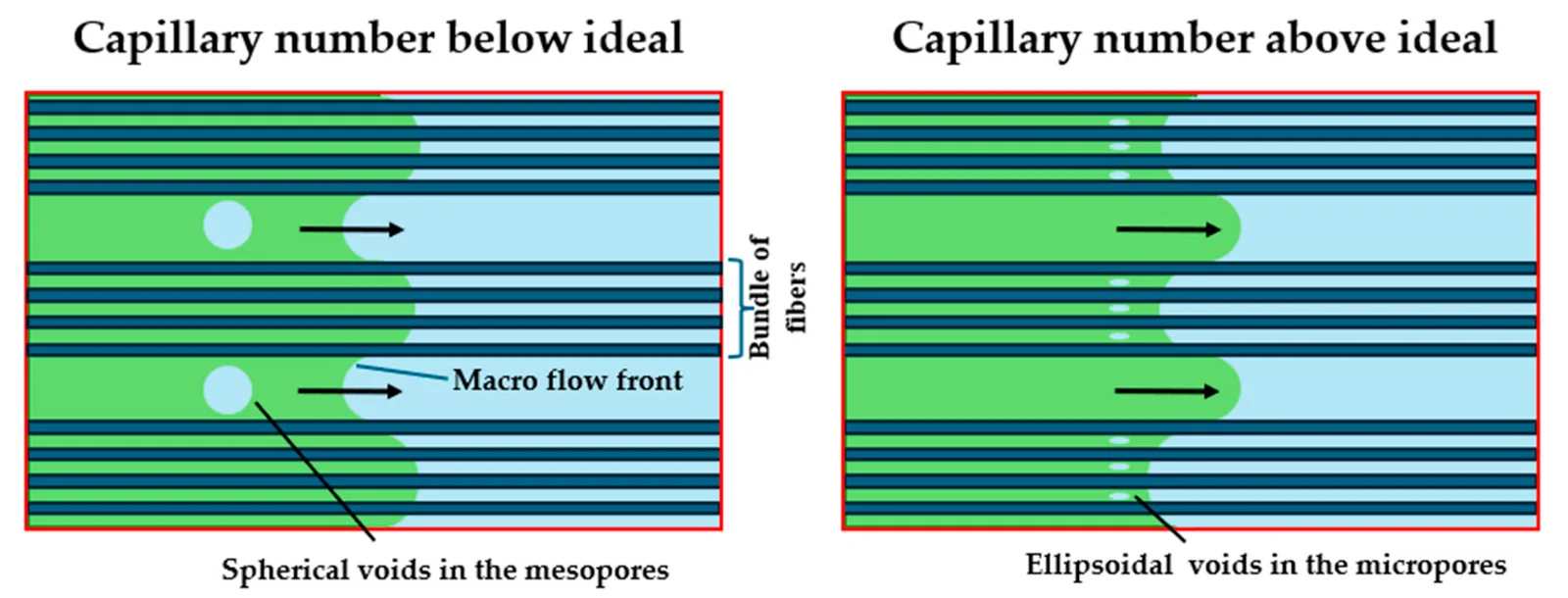
Representation of the capillary number effect on the void formation during composite fabrication using the RTM process.

For composite production, the viscous and capillary effects must be balanced, resulting in better filling of voids in the fibrous medium and minimizing air entrapment [23]. This balance creates an ideal capillary number that varies with the polymer type and the manufacturing process [24,25]; it ranges from 0.5 × 10^−3^ to 1.7 × 10^−3^ [26,27,28]. When the capillary number is lower than the ideal (due to a low impregnation speed), there is a tendency for spherical voids to form in the inter-tow, while when the capillary number is higher than the ideal (due to a high impregnation speed), there is a tendency for ellipsoidal voids to form in the intra-tow [29,30], as shown in Figure 1. In production processes with constant injection pressure, the speed of the flow front drops as the impregnated length increases [23], generating a capillary number field inside the mold.

Conversely, an examination of the actual literature of studies related to computer simulations and modelling applied to composite manufacturing by the RTM process reveals a predominance of studies employing simplified models. These models frequently assume isothermal, resin-only (single-phase flow), neglect the effects of air entrapment within the mold, and consider constant resin viscosity [31,32,33,34]. Such assumptions tend to align the numerical results closely with those predicted by simplified analytical models.

A smaller group of studies incorporates the effects of the resin–air stream (multiphase flow) into the mathematical models, increasing the accuracy of numerical simulations in representing the actual phenomenon, although they still assume constant resin viscosity [35,36,37]. Considering multiphase flow enables the analysis of resin–air interactions, allowing, for instance, the identification of potential air entrapment points within the composite. This approach, consequently, represents a more refined and sophisticated analysis.

A limited number of investigations introduce an additional complexity into the modeling beyond multiphase flow: the consideration of non-isothermal flow [38]. Incorporating the energy equation into the model enables the evaluation of new process parameters, including the mold and resin temperature during impregnation. This approach allows for the improvement of production processes and enhances the understanding of resin curing dynamics, as well as the impact of temperature gradients within the thermal system on the properties of the composite.

Intermediate modeling approaches also exist, in which non-isothermal single-phase flow is considered, with resin viscosity treated as either variable [39,40,41,42] or constant [43,44]. These models enable the assessment of thermal effects in the process without introducing the numerical and physical complexities of multiphase flow, but they provide a less detailed representation of the overall process phenomena.

Finally, initial research has also explored a macroscopic approach, using the simplest models, with isothermal and single-phase flow, with constant injection pressure and fluid properties [8,9,45] or using a more complex model, with non-isothermal and multiphase flow (vegetable oil and air), with variable injected fluid viscosity and injection pressure [46], to examine the influence of the resin absorption by the micropores of the fibers. Resin impregnation into the fibers reduces penetration depth and delays the stabilization of pressure, highlighting the importance of improving fiber–resin interactions within the sealed mold. Despite its significance, this phenomenon remains crucial to incorporate resin absorption effects into more sophisticated models, particularly with the use of advanced CFD techniques, to enhance and deepen the understanding of the RTM process. The impact of this sink term depends on fiber architecture: for instance, when resin flows along the fiber-to-fiber direction, it tends to move through the channels between fibers rather than fully impregnating them; conversely, when flow occurs perpendicular to the direction of the fibers, the resin distributes more uniformly, with a smaller delay effect and less influence from pressure drops at the inlet.

Based on the above considerations, this research seeks to contribute numerically (using CFD tools) to the composite manufacturing by RTM, advancing the complexity of the analysis, using a non-isothermal and multiphase flow (polymeric resin and air), with variable resin viscosity and constant injection pressure or injection volumetric flow rate, and including the absorption of resin by the fiber micropores, based on an equation dependent on the mold temperature, evaluating, in a way that is unprecedented in this context, the capillary number field to obtain information about the potential presence of voids in this product.

The numerical and methodological model applied to the RTM process in this study is innovative because it encompasses different themes: (a) implements epoxy resin injection within the most advanced numerical model currently available in the literature [46], thereby bringing the simulations closer to real manufacturing processes; (b) it employs a viscosity model for the resin that indirectly accounts for the effects of curing throughout the injection process, and (c) the capillary number field is used to extract information regarding the potential formation of voids in the manufactured composite, based on the injection operating conditions. The idea is to support researchers and engineers in achieving a better understanding and more accurate modeling of the physical phenomena governing the RTM composite manufacturing process, particularly in radial systems, with a focus on the capillary number and the evaluation of injection conditions (constant pressure vs. constant volumetric flow rate).

## 2. Methodology

### 2.1. Problem Description and Mold Shape

This paper presents a theoretical investigation of resin flow through a porous medium confined within a heated and closed mold. The resin is introduced at a gate located at the center of the mold and propagates radially toward the outlet gates at the corners, while exchanging heat with both the reinforcement and the mold walls.

**Figure 2:**
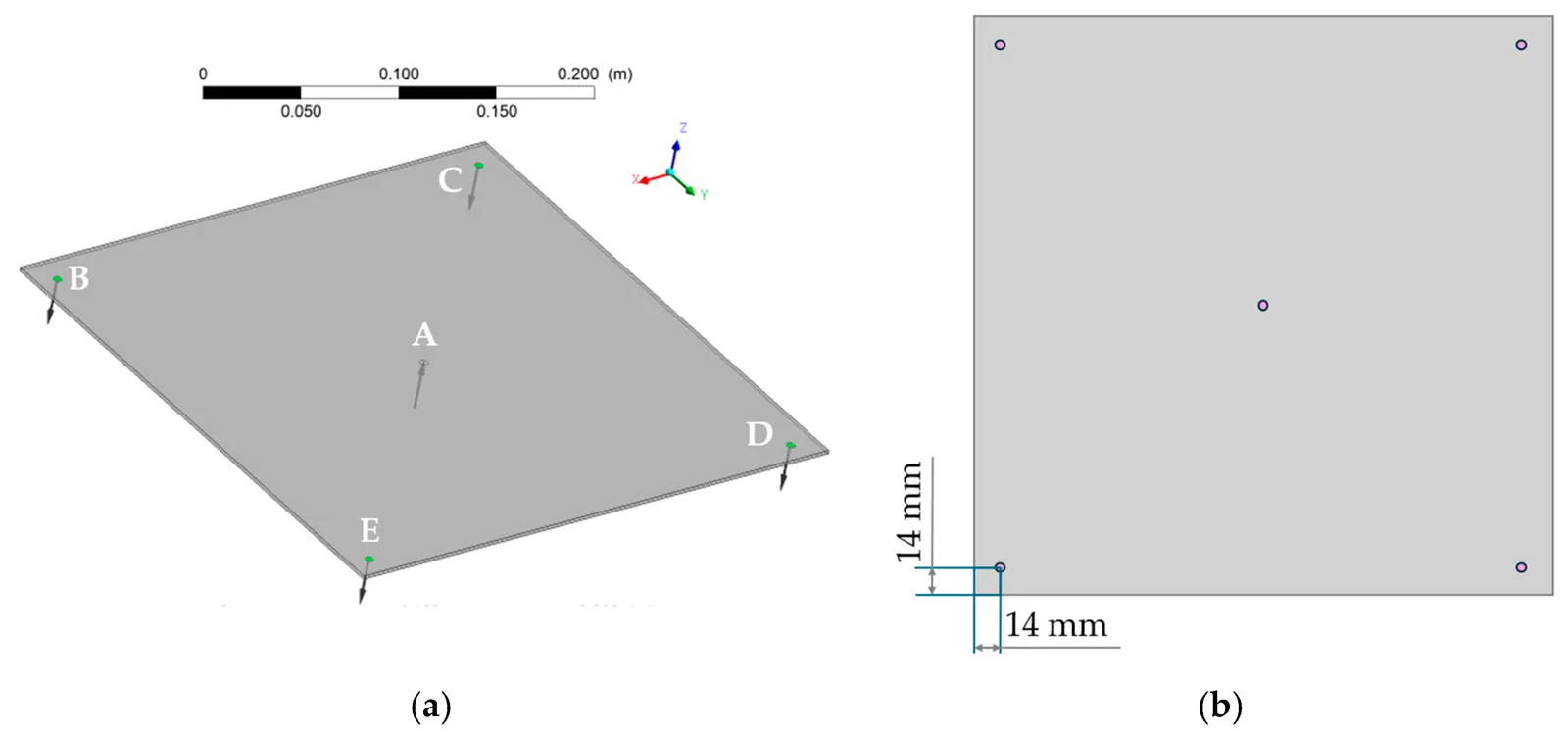
Mold geometry. (**a**) Perspective view, with a focus on the injection gate (A) and the exit gates (B–E), and (**b**) the bottom view [46].

The mold under analysis has a parallelepipedal shape with a square base measuring 300 × 300 mm^2^ and a thickness of 2 mm. The injection point is located at the center, while four symmetric outlet points are placed near the corners, as shown in Figure 2. Both the inlet and outlets are circular holes with a diameter of 4.58 mm, positioned at the bottom surface. In this configuration, resin is injected upward, whereas the outlets allow flow in the downward direction.

### 2.2. Numerical Grid

**Figure 3:**
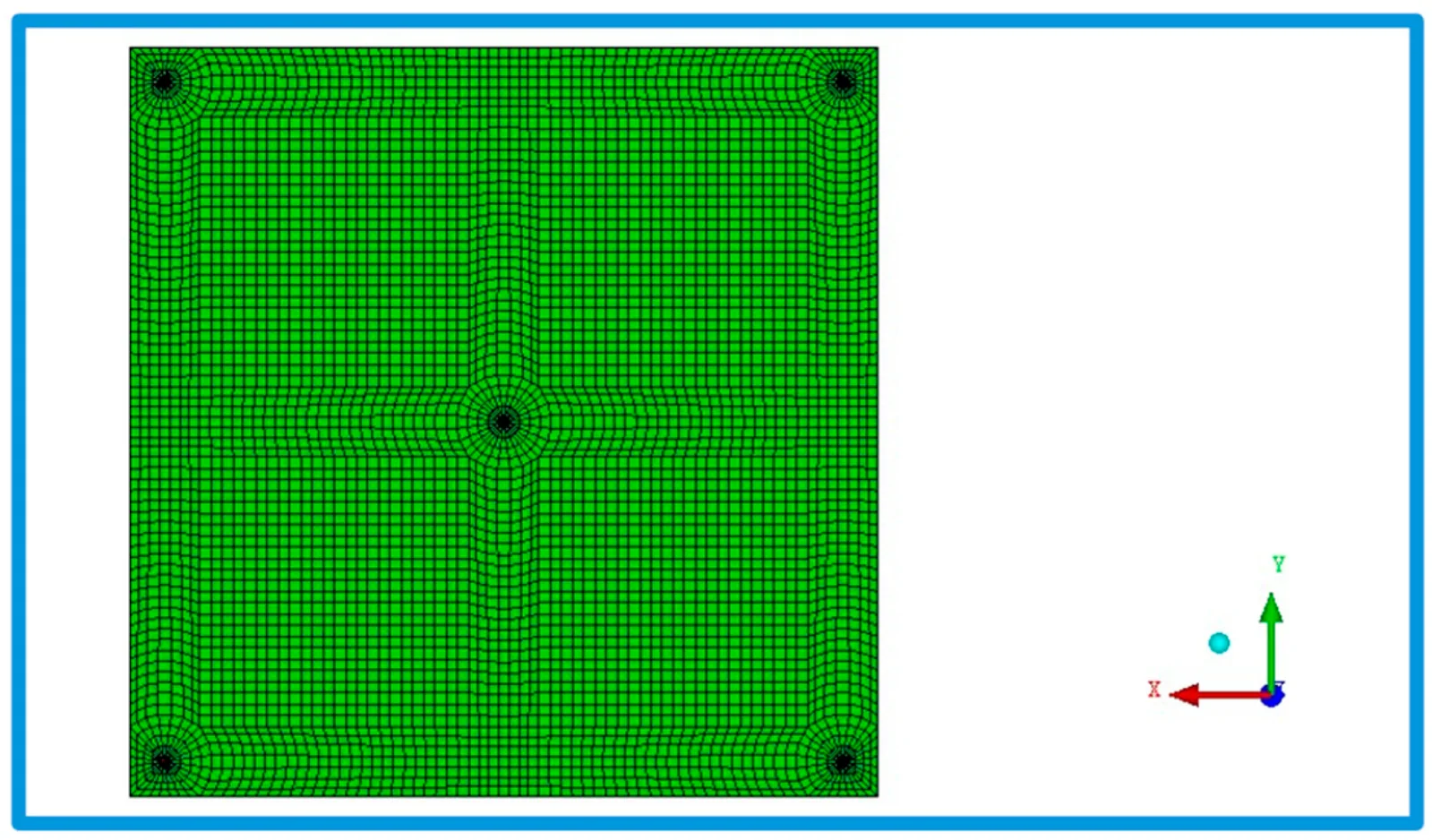
Bottom view of the grid [46].

**Figure 4:**
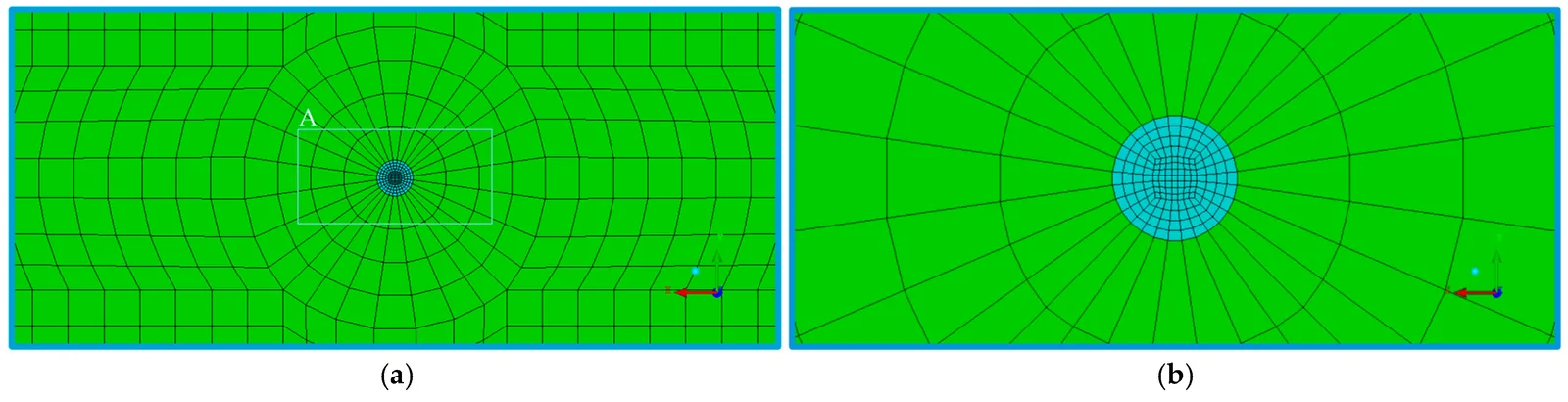
Injection point placed near the lower boundary of the computational mesh (**a**), with detailed view ‘A’ illustrating the injection region (**b**) [46].

**Figure 5:**
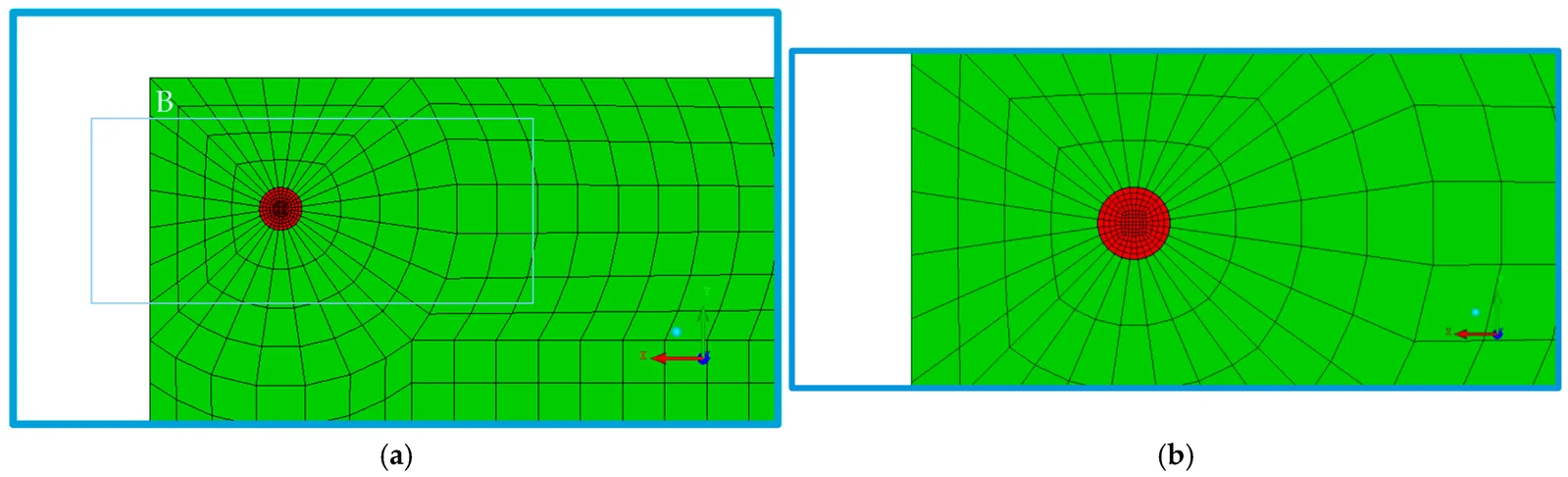
View of one of the four outlet points located near the lower region of the computational mesh (**a**), with detail ‘B’ providing close-ups of this outlet (**b**) [46].

**Figure 6:**
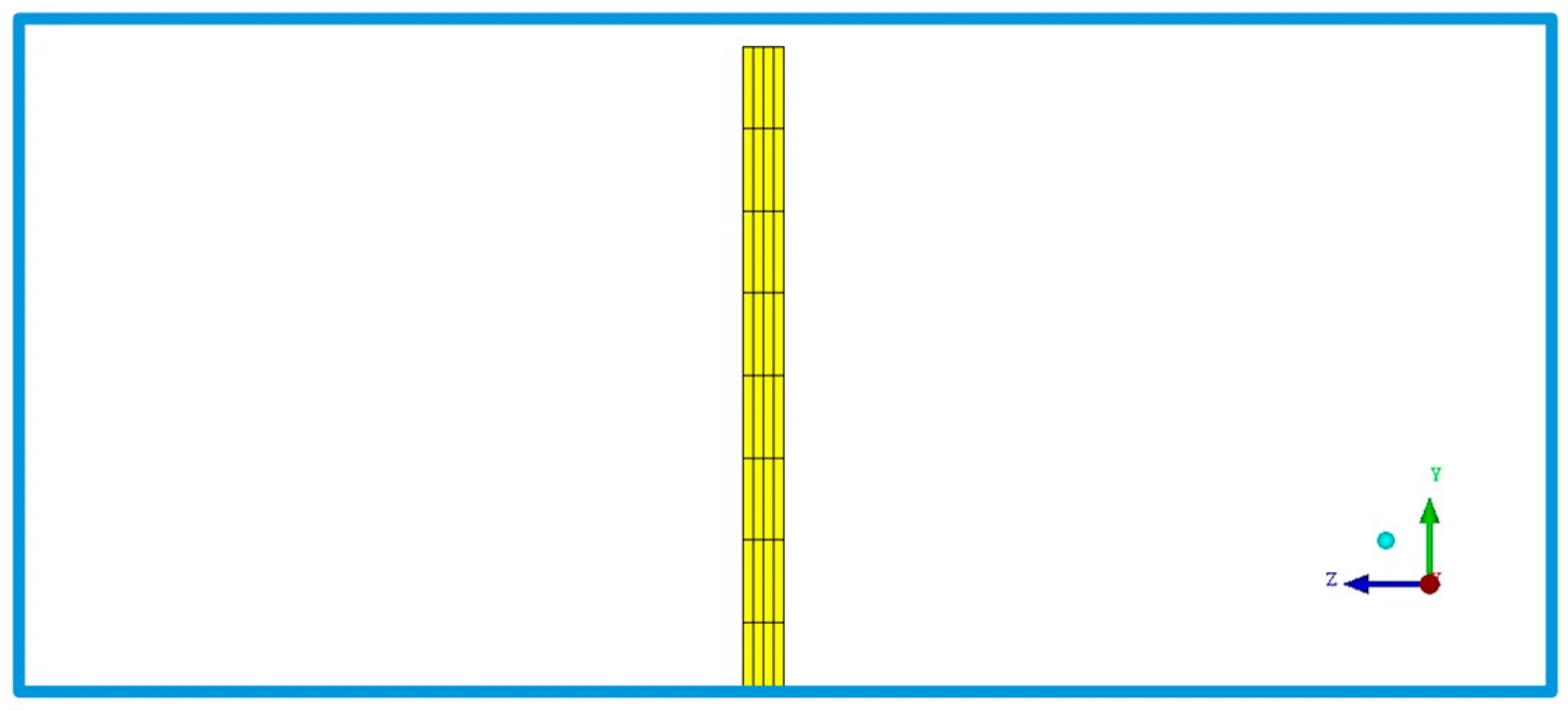
Side view of the grid [46].

To investigate the effect of grid refinement on the theoretical results, the Grid Convergence Index (GCI) technique was utilized. This technique, widely adopted in previous studies [47,48], provides an estimate of the discretization errors associated with the refinement level of the computational mesh [49]. As a result of this procedure, an optimized structured mesh was obtained, consisting of 22,852 hexahedral elements and 29,260 nodes, as shown in Figure 3, Figure 4, Figure 5 and Figure 6, with a numerical uncertainty of 0.17%, which was considered satisfactory. The mesh was generated using Ansys ICEM CFD (v. 2021 R2), while the numerical simulations were performed with Ansys CFX (v. 2021 R2). Ansys CFX software was chosen over specialized RTM programs, such as PAM-RTM, due to its broader selection of numerical models and its flexibility in modifying or implementing the governing differential equations of the physical phenomena. For the simulations, the High Resolution advection scheme and Second Order Backward Euler transient scheme were used. The convergence criterion was 1 × 10^−4^ Root Mean Square (RMS), with a maximum of 10 iterations per time step. The time step used in the simulations was 0.5 s, defined after sensitivity analysis.

### 2.3. Theoretical Modeling

#### 2.3.1. The Balance Equations

In order to analyze the multiphase flow of the resin and air within a heated mold filled with a fibrous preform during the RTM process, the following assumptions were used:

(a) Three-dimensional, transient, laminar, and non-isothermal flow;
(b) The fluids flowing inside the porous media are incompressible;
(c) The fibrous medium is isotropic, which is valid considering reinforcement arranged in 0/90° architecture;
(d) Energy generation within the porous media and fluids is neglected;
(e) Resin dynamic viscosity is variable during the process and considers the curing effect;
(f) The resin absorption by the reinforcement is taken into account.

**Figure 7:**
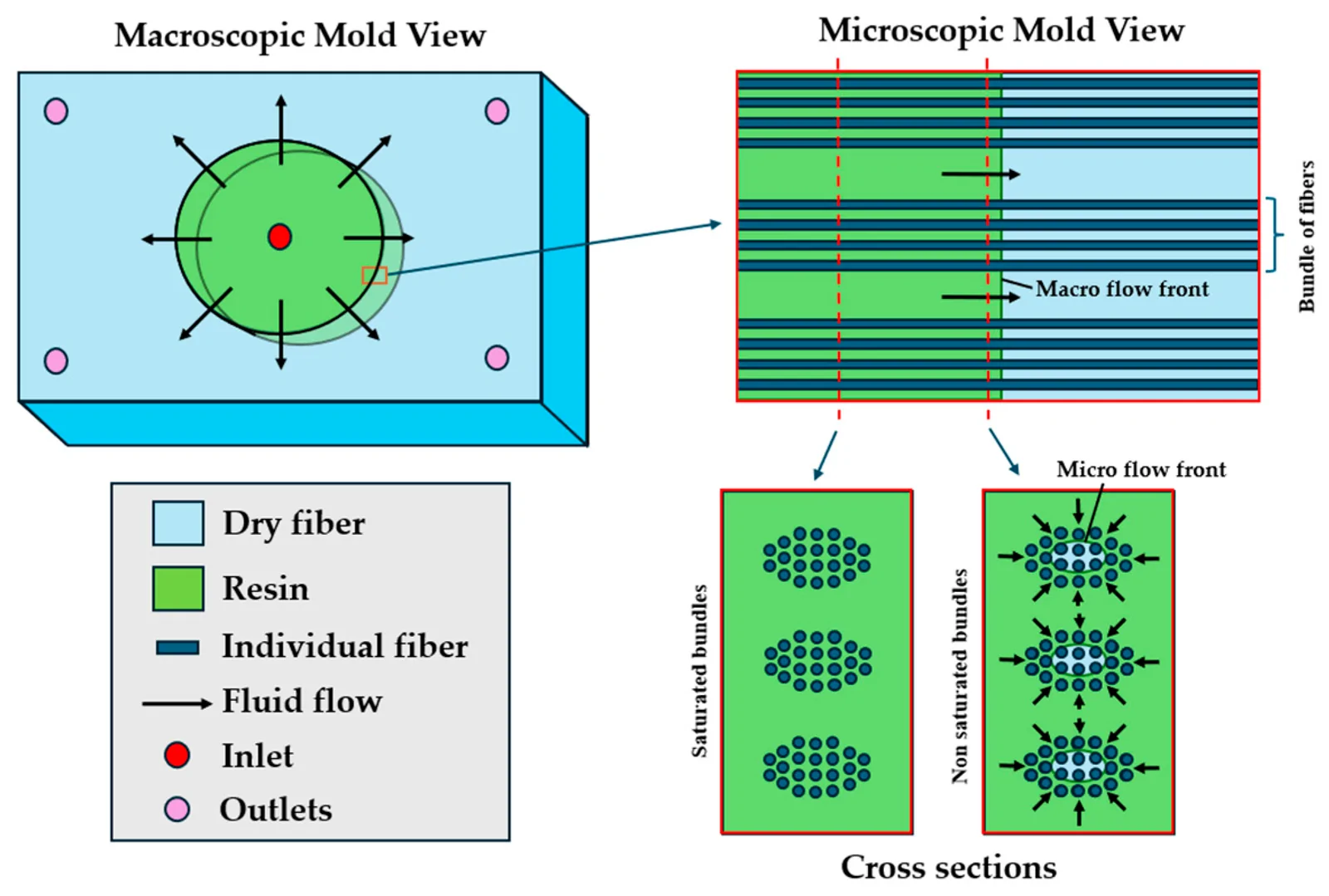
Representative scheme of resin flow into the mold, highlighting the resin absorption by the fibrous reinforcement [46].

Figure 7 shows the infiltration process of resin into the micropores of the bundles of reinforcement fiber yarns. To predict the resin behavior within the porous medium during the infiltration process, the mass, momentum, and energy equations were applied, adopting a homogeneous multiphase model, i.e., a single velocity field for resin and air. The fundamental equations are presented below:

(a) Mass balance equation:

The mass balance equation, as applied to the resin injected into the mold filled with fibers, can be expressed by symbols as follows:

(2) $$\frac{\partial }{\partial t}\left(f_{r}\varphi \rho_{r}\right)+\nabla \cdot \left({f_{r}\rho }_{r}{\vec{U}}_{m}\right)=-f_{r}S^{M}=-f_{r}s\rho_{r}$$

where *φ* signifies the porosity of the porous medium; *f_r_* is the volumetric fraction of the resin; *ρ_r_* is the density of the resin; *U_m_* is the surface velocity vector of the fluids; *S^M^* is the source term; and *s* represents the resin mass flow rate per unit mass of that fluid absorbed by the porous medium.

Based on the experimental results reported in the literature [50,51,52], numerical simulations were performed to minimize the error between the predicted and experimental data, in order to obtain the parameter s for each operating condition. After this procedure, a fitting equation for the source term, *s*, expressed in s^−1^, as a function of the effective resin viscosity at the mold temperature, $\mu_{ef}$, between 32 and 276 cP, and mold temperature ranging from 30 and 60 °C, was obtained (*R*^2^ = 100.00%) and is shown below:

(3) $$s=\left[\left(3.332\times e^{-0.115\times \frac{\mu_{ef}}{cP}}\right)\times 10^{-4}\right]{\;s}^{-1}$$

The general mass conservation equation applied to the air flowing within the mold will be:

(4) $$\frac{\partial }{\partial t}\left(f_{a}\varphi \rho_{a}\right)+\nabla \cdot \left({f_{a}\rho }_{a}{\vec{U}}_{m}\right)=0$$

where *f_a_* is the air volumetric fraction, and *ρ_a_* is the air density.

The volumetric fractions of the resin and air, at any location within the fibers, can be written as:

(5) $$f_{r}+f_{a}=1$$

(b) Linear momentum balance equation:

The linear momentum conservation equation is given by:

(6) $$\frac{\partial }{\partial t}\left(\varphi \rho_{m}{\vec{U}}_{m}\right)+\nabla \cdot \left(\rho_{m}{\vec{U}}_{m}⊗{\vec{U}}_{m}\right)-\nabla \cdot \left[{µ}_{m}\left(\nabla {\vec{U}}_{m}+{\left(\nabla {\vec{U}}_{m}\right)}^{T}-\frac{2}{3}\delta \nabla \cdot {\vec{U}}_{m}\right)\right]=\varphi \vec{S^{\alpha }}-\varphi \nabla p$$

where *ρ_m_* is the weighted density of the fluids, *µ_m_* is the weighted dynamic viscosity of fluids (resin and air), *δ* is the second-order identity tensor, *S^α^* is the source term of linear momentum, and *p* is the pressure, including the gravitational effect.

The fluid-weighted density can be determined by:

(7) $$\rho_{m}={f_{r}\rho }_{r}+{f_{a}\rho }_{a}$$

The fluid-weighted dynamic viscosity can be determined by:

(8) $${µ}_{m}={f_{r}µ}_{r}+{f_{a}µ}_{a}$$

where *µ_r_* is the dynamic viscosity of the resin and *µ_a_* is the dynamic viscosity of the air.

The linear momentum source term, *S^α^*, accounts in the model for the loss of motion due to the yield strength imposed by the porous medium, and is expressed as follows:

(9) $$\vec{S^{\alpha }}=-\frac{{µ}_{m}}{K}{\vec{U}}_{m}-K_{loss}\frac{\rho_{m}}{2}\left|{\vec{U}}_{m}\right|{\vec{U}}_{m}$$

where *K_loss_* is the quadratic moment loss coefficient, and *K* is the permeability of the porous medium. In this study, the second term is negligible due to the low velocities of the flow under investigation.

(c) Energy Balance Equation:

To consider the thermal effects in the study, the energy balance must be added to the conservation equations system. In this approach, a non-homogeneous multiphase flow is considered, meaning that different temperature fields are assigned to each fluid phase and to the porous medium (solid). Under these conditions, the energy conservation equation is expressed as follows.

For the resin:

(10) $$\frac{\partial }{\partial t}\left({f_{r}\varphi \rho }_{r}h_{r}\right)+\nabla \cdot \left({f_{r}\rho }_{r}{\vec{U}}_{m}h_{r}\right)-\nabla \cdot \left({f_{r}k}_{r}\nabla T_{r}\right)=Q_{rp}+Q_{ra}$$

where *h_r_* is the enthalpy per mass unit of the resin, *k_r_* is the thermal conductivity of the resin, *T_r_* is the temperature of the resin, $Q_{rp}$ is the interfacial heat transfer to the resin from the porous medium, and $Q_{ra}$ is the interfacial heat transfer to the resin from the air.

For the air:

(11) $$\frac{\partial }{\partial t}\left({f_{a}\varphi \rho }_{a}h_{a}\right)+\nabla \cdot \left({f_{a}\rho }_{a}{\vec{U}}_{m}h_{a}\right)-\nabla \cdot \left({f_{a}k}_{a}\nabla T_{a}\right)=Q_{ap}-Q_{ra}$$

where *h_a_* is the specific enthalpy of the air, *k_a_* is the air thermal conductivity, and *T_a_* is the air temperature, and $Q_{ap}$ is the interfacial heat transfer to the air from the porous medium.

For the porous medium:

(12) $$\frac{\partial }{\partial t}\left[{\left(1-\varphi \right)\rho }_{s}{C_{s}T}_{s}\right]-\nabla \cdot \left[\left(1-\varphi \right)k_{s}\nabla T_{s}\right]={-Q}_{rp}-Q_{ap}$$

where *ρ_s_* is the density, *C_s_* is the specific heat, *k_s_* is the thermal conductivity, and *Ts* is the temperature of the porous medium.

The Reynolds number is a fundamental dimensionless parameter used to describe flow regimes, representing the ratio of inertial to viscous forces acting on a fluid. For flow within a porous medium, this dimensionless parameter can be written as [53]:

(13) $${Re}_{pm}=\frac{\rho_{r}\;\times \;U\;\times \;\sqrt{K}}{{µ}_{r}}$$

where *Re_pm_* is the Reynolds number for flow in porous media, and *U* is the surface velocity of the resin in the direction of flow.

It is important to note that it was not required to include governing equations for turbulence in the proposed model, because the Reynolds number for flow in porous media, calculated using Equation (13), is much less than 1, indicating that the flow is laminar (Darcy regime).

It should be noted that the mathematical model presented here is the same one reported by a previous study [46], already verified and validated by these authors.

#### 2.3.2. Boundary Conditions

The domain is subject to the following boundary conditions (Figure 2):

(a) Injection gate (point A):

The resin is injected at a temperature of 23 °C and a prescribed gauge pressure of 2.0 bar or a prescribed volumetric flow rate of 3.5 × 10^−8^ m^3^/s. This volumetric flow rate value was defined because, for the operating conditions simulated in this research, it provides average pressure levels close to 2.0 bar, allowing a qualitative comparison between the results obtained under both conditions.

(b) Outlet gate (points B–E):

Null prescribed gauge pressure.

(c) Walls of the mold:

Non-slip hypothesis (zero fluid velocity), with a prescribed temperature ranging from 30 to 60 °C.

(d) Fibrous medium:

The prescribed initial temperature ranges from 30 to 60 °C, and the medium is fully filled with initially static air (zero velocity), with a specified initial temperature equal to that of the porous medium (i.e., 30 to 60 °C).

### 2.4. Materials Properties

The fiber preform analyzed is a commercial flat fabric made of E-glass fiber with a 0/90° orientation (300 g/m^2^), whose properties are listed in Table 1. The source term (mass flow rate of resin, per unit mass of resin absorbed by the fiber), *s*, is shown in Table 2.

The fluid injected into the mold was epoxy resin, used to manufacture aeronautical composites using the RTM technique. The properties of this material are described in Table 3. The dynamic viscosity of the resin follows an empirical function, based on the Dual-Arrhenius viscosity model given below [55]:

(14) $$\mu_{r}=1.1380\times 10^{-11}e^{\left(\frac{7248.5623}{T}+1.587\times 10^{9}t\times e^{\left(\frac{-7735.0037}{T}\right)}\right)}$$

where *μ_r_* is the resin dynamic viscosity (kg/m·s), *T* is the temperature of the fluid (K), and *t* is the time (min). It is important to note that the time parameter in Equation (14) indirectly accounts for the effect of resin curing in the viscosity model. As the injection progresses, the increase in time leads to an increase in resin viscosity, thereby indirectly capturing the progressive curing of the resin. This behavior reflects the increase in the cure rate of the resin during the injection process and its consequent effect on the flow behavior.

The properties of the air enclosed in the mold at the initial stage of the resin injection are summarized in Table 4.

## 3. Results and Discussion

### 3.1. Verification and Validation

In order to achieve a high degree of reliability in the results obtained by the developed numerical-mathematical model, the V&V (Verification and Validation) procedure was executed. For all these numerical cases, the same numerical grid and mathematical modeling already presented were used, with boundary conditions and material properties similar to those used in the cases under comparison.

Verification consists of comparing the numerical results with known analytical and highly accurate numerical solutions, aiming to determine whether the computational implementation is correct [61]. For verification, the time at which the injected fluid touches the mold walls obtained from a numerical case available in the literature [45], *t_b_*, was compared with the time obtained using the model developed in the present work, *t_bv_* (Table 5). It is noteworthy that the case evaluated in the literature [45] uses the same resin absorption source-term concept as that employed in the present work, but with an unheated mold and a single-phase, one-dimensional flow. A deviation of less than 2% was observed, indicating an excellent approximation between the evaluated parameters.

Validation consists of comparing numerical results with experimental results, aiming to verify whether the physical models employed in the code are consistent with the physical reality of the problem addressed [61]. For validation, the time at which the injected fluid touches the mold walls obtained from experimental cases available in the literature [50], *t_b_*, was again compared with the time obtained using the model developed in the present work, *t_bv_* (Table 5). A deviation of less than 5% was observed, indicating, once again, an excellent approximation between the evaluated parameters.

**Figure 8:**
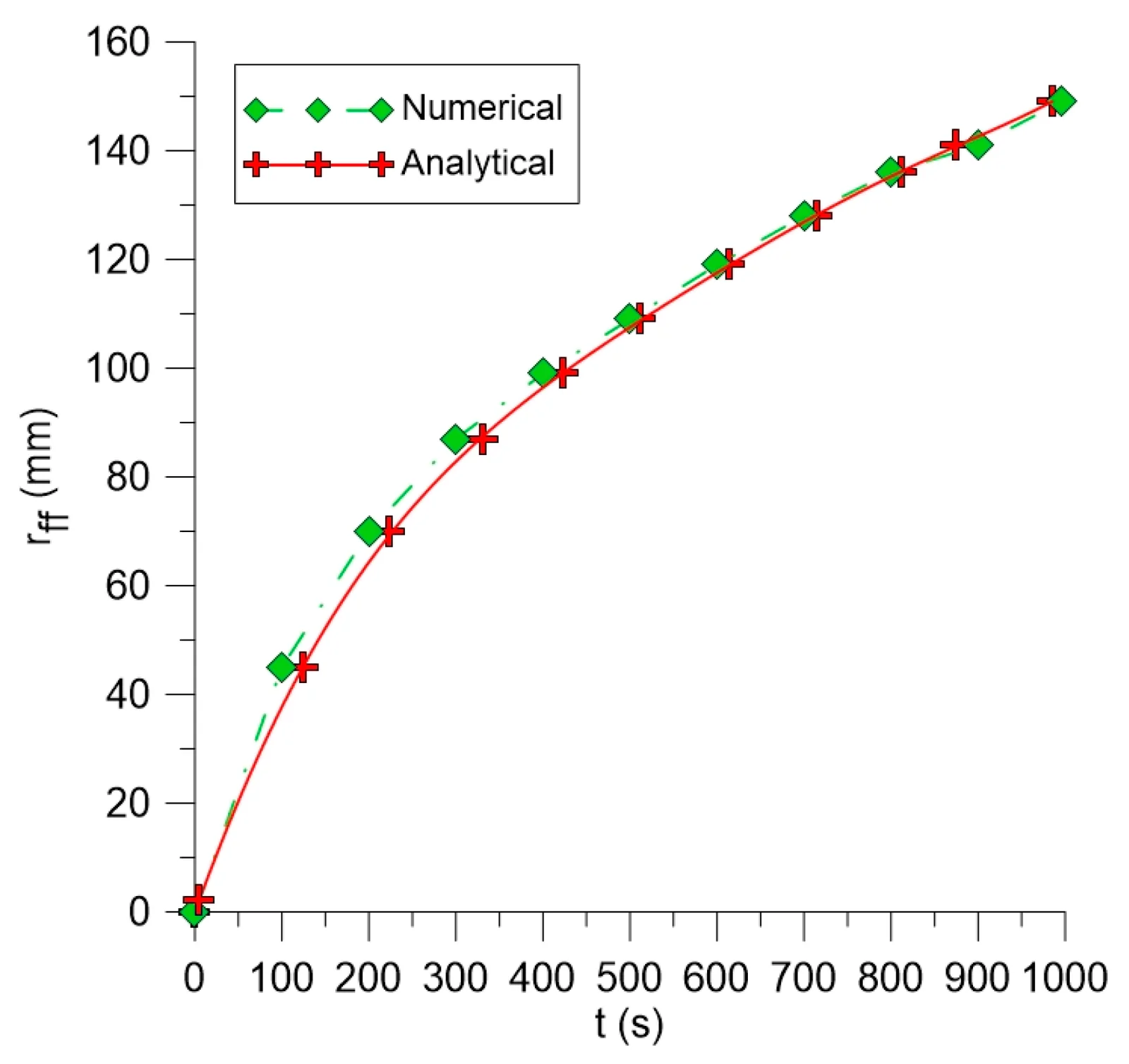
Comparison of the flow front evolution over time between the numerical simulation (isothermal injection) and the analytical solution (Equation (15)), for s = 0 s^−1^.

Additionally, for verification purposes, the position of the flow front, $r_{ff}$, as a function of time, t, was compared with the analytical solution obtained from Equation (15) (valid for single-phase, isothermal, isotropic, 2D axisymmetric radial flow and s = 0 s^−1^ [10]) and the numerical solution obtained from the developed model (Figure 8). A good agreement between the curves was observed, with deviations of less than 10% for radial positions greater than 87 mm.

(15) t=μrφ2Kpinjrff2lnrffrinj−0.5rff2−rinj2

where *μ_r_* is the resin dynamic viscosity (adopted as 58.6 cP), φ is the porosity of the reinforcement (adopted as 0.5829), K is the permeability of the reinforcement (adopted as 5.24 × 10^−11^ m^2^), $p_{inj}$ is the injection pressure (adopted as 27,013 Pa) and $r_{inj}$ is the injection channel radius (adopted as 2.29 mm). It is worth noting that the property values described in this section were used only in V&V analyses.

**Figure 9:**
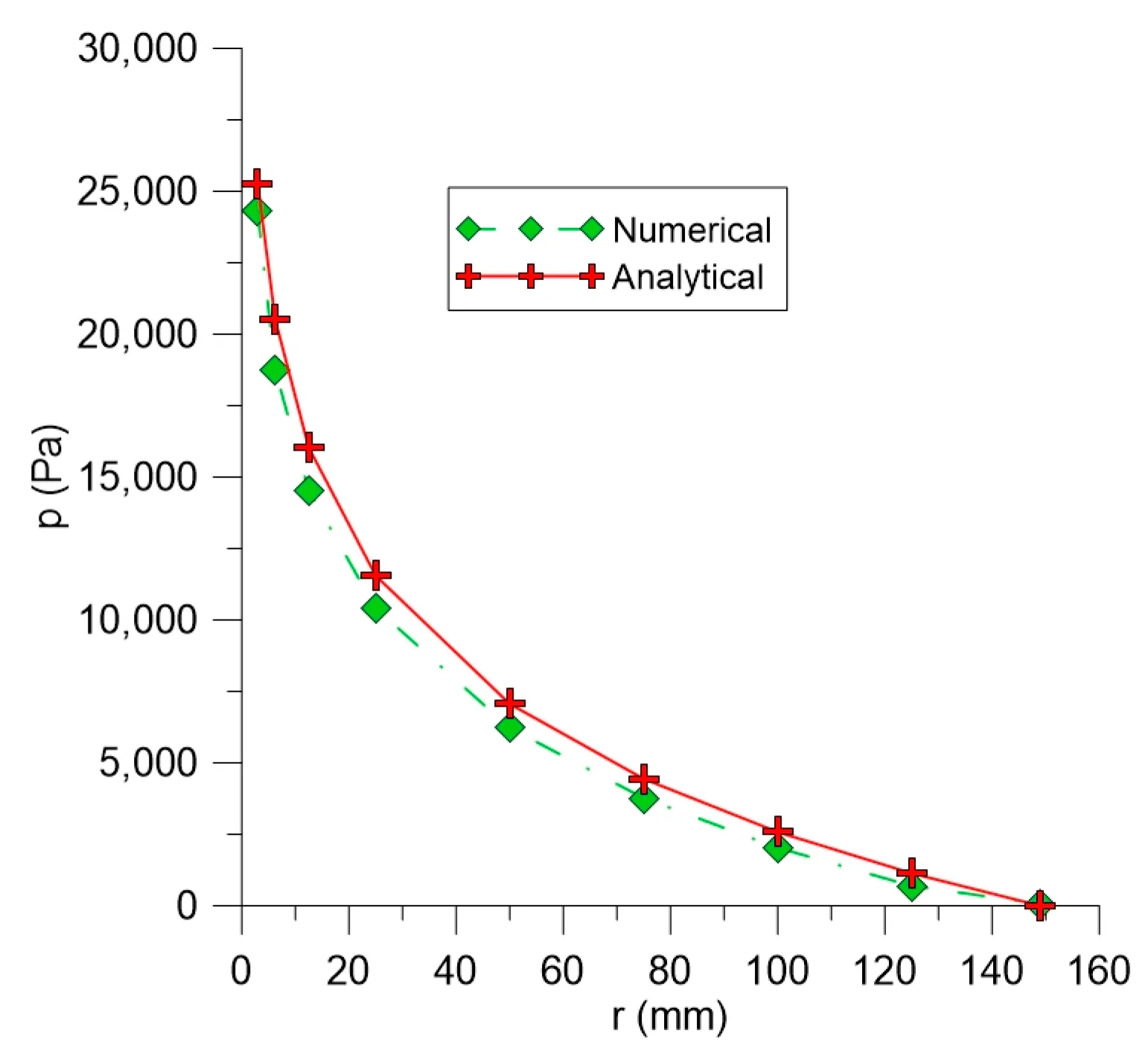
Comparison of the pressure profile between the numerical simulation (isothermal injection) and the analytical solution (Equation (16)), for s = 0 s^−1^.

Still in the analytical verification phase, the pressure profile, p, along the mold centerline (at a thickness of 1 mm), versus radial position, r, was compared with the analytical solution obtained from Equation (16) (valid for single-phase, isothermal, isotropic, 2D axisymmetric radial flow and s = 0 s^−1^ [45]) and the numerical solution obtained from the developed model, at a simulation time of 995 s (Figure 9), which corresponds to the moment when the resin touches the mold walls ($r_{ff}$ = 150 mm). Again, a good agreement between the curves was observed, with deviations of less than 10% for radial positions below 25 mm.

(16) p=pinjlnrrfflnrinjrff

**Figure 10:**
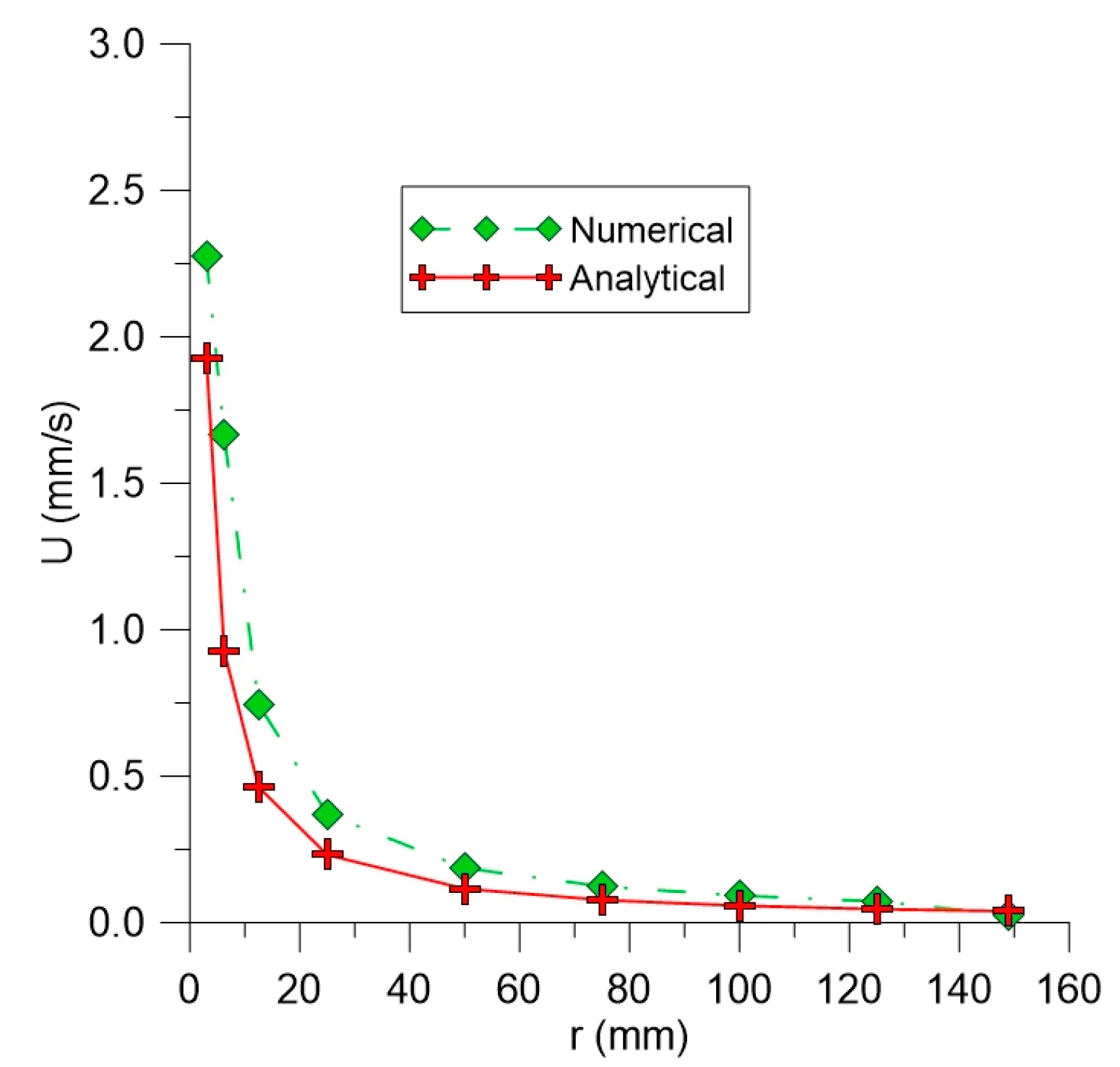
Comparison of the resin velocity profile between the numerical simulation (isothermal injection) and the analytical solution (Equation (17)), for s = 0 s^−1^.

Subsequently, in the analytical verification, the resin velocity profile, U, along the mold centerline (at a thickness of 1 mm), versus radial position, r, was compared with the analytical solution obtained from Equation (17) (valid for single-phase, isothermal, isotropic, 2D axisymmetric radial flow and s = 0 s^−1^ [45]) and the numerical solution obtained from the developed model, at a simulation time of 995 s (Figure 10), which corresponds to the moment when the resin touches the mold walls ($r_{ff}$ = 150 mm). Once again, even with the differences in the assumptions adopted in the analytical and numerical models, a good agreement between the curves was verified.

(17) U=−Krμpinjlnrinjrff

**Figure 11:**
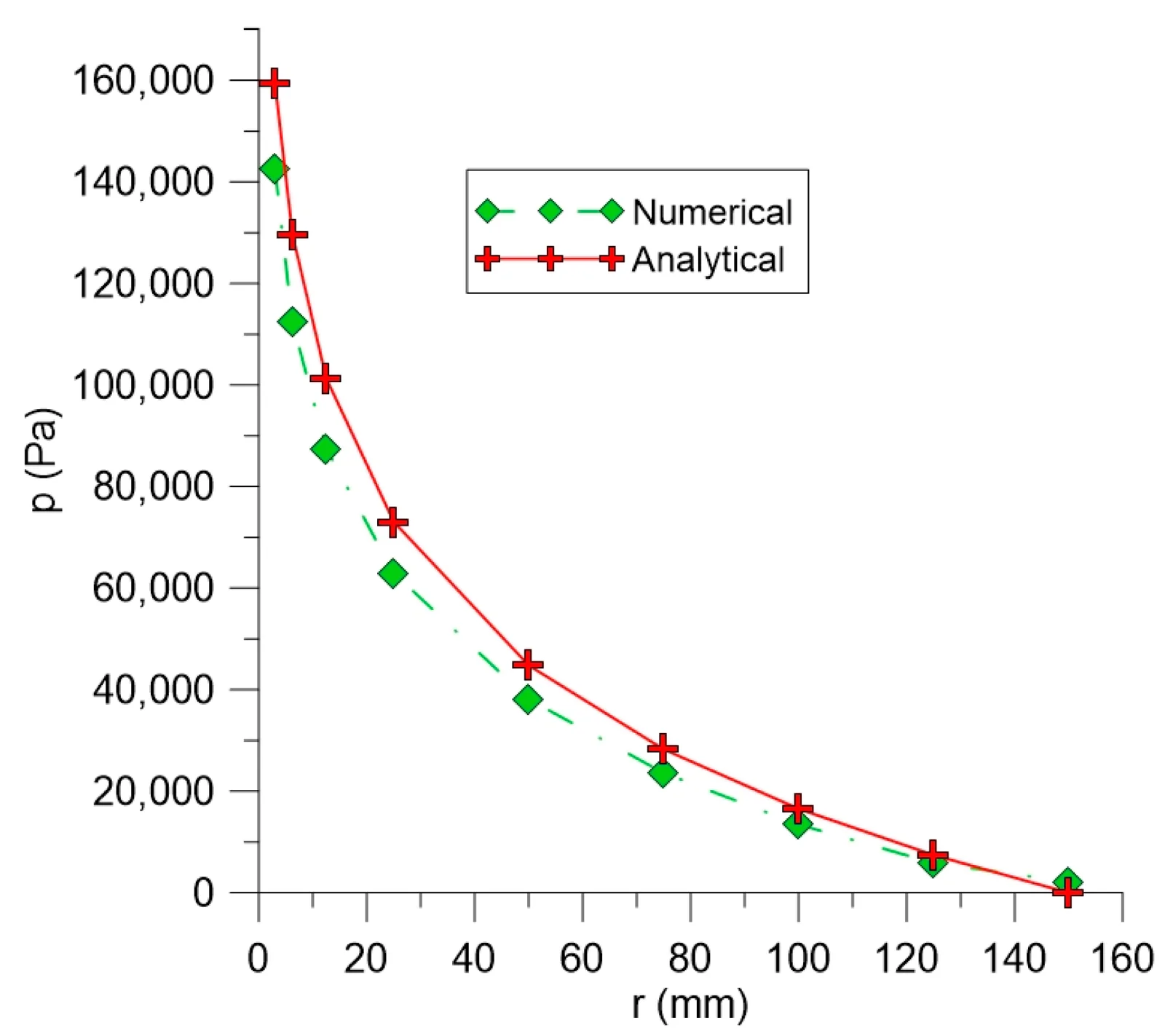
Comparison of the pressure profile between the numerical simulation (isothermal injection) and the analytical solution (Equation (18)), for s > 0 s^−1^.

Next, the pressure profile, p, along the mold centerline (at a thickness of 1 mm), versus radial position, r, was compared with the analytical solution obtained from Equation (18) (valid for single-phase, isothermal, isotropic, 2D axisymmetric radial flow and s > 0 s^−1^ [45]) and the numerical solution obtained from the developed model, at a simulation time of 570 s (Figure 11), which corresponds to the moment when the resin touches the mold walls ($r_{ff}$ = 150 mm). The numerical and analytical results showed good agreement, with deviations of less than 20% for radial positions below 100 mm.

(18) p=sμr4Kr2−rff2+pinj+sμr4Krff2−rinj2lnrrfflnrinjrff

where *s* is the resin absorption term (adopted as 1 × 10^−7^ s^−1^), *μ_r_* is the resin dynamic viscosity (adopted as 58.6 cP), K is the permeability of the reinforcement (adopted as 1.48 × 10^−11^ m^2^), $p_{inj}$ is the injection pressure (adopted as 170,479 Pa) and $r_{inj}$ is the injection channel radius (adopted as 2.29 mm).

**Figure 12:**
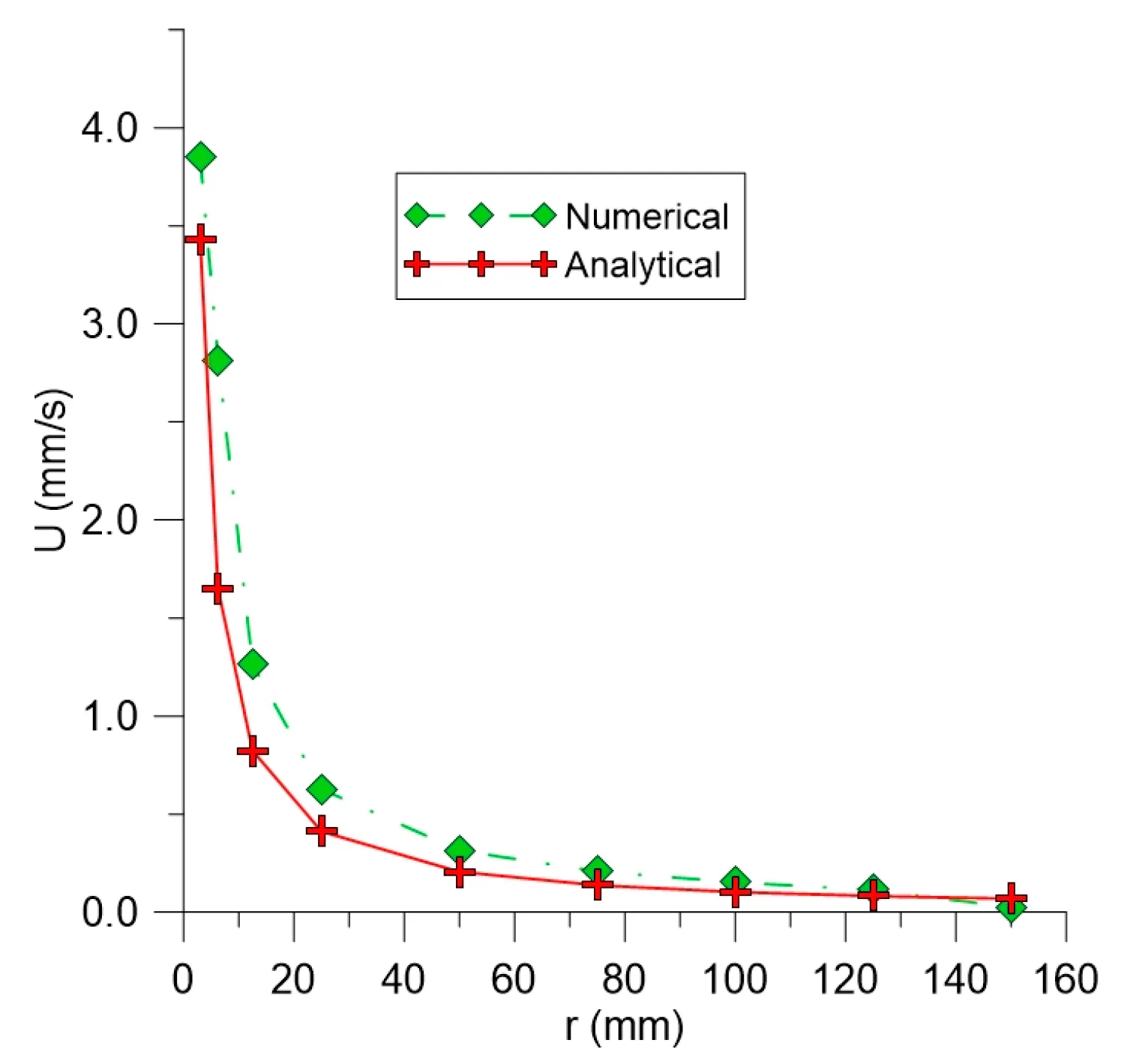
Comparison of the resin velocity profile between the numerical simulation (isothermal injection) and the analytical solution (Equation (19)), for s > 0 s^−1^.

Finally, in the verification phase, the resin velocity profile, U, along the mold centerline (at a thickness of 1 mm), versus radial position, r, was compared with the analytical solution obtained from Equation (19) (valid for single-phase, isothermal, isotropic, 2D axisymmetric radial flow and s > 0 s^−1^ [45]) and the numerical solution obtained from the developed model, at a simulation time of 570 s (Figure 12), which corresponds to the moment when the resin touches the mold walls ($r_{ff}$ = 150 mm). The comparison revealed good agreement between the curves.

(19) U=−Kμrsμr2Kr+pinj+sμr4Krff2−rinj2lnrinjrff1r

It should be emphasized that the analytical solutions considered for comparison are applicable to simpler conditions than those adopted in the numerical model, namely, single-phase and 2D axisymmetric radial flow, with variations only along the radial coordinate r, and without considering the influence of the mold walls on the flow. Therefore, some deviations between the analytical and numerical results are expected. Considering these differences in the underlying assumptions, the agreement between the analytical and numerical results was considered satisfactory for verification purposes. Based on the results obtained from the V&V analyses, the developed numerical model was considered reliable.

### 3.2. Evaluation of the Resin Flow

**Figure 13:**
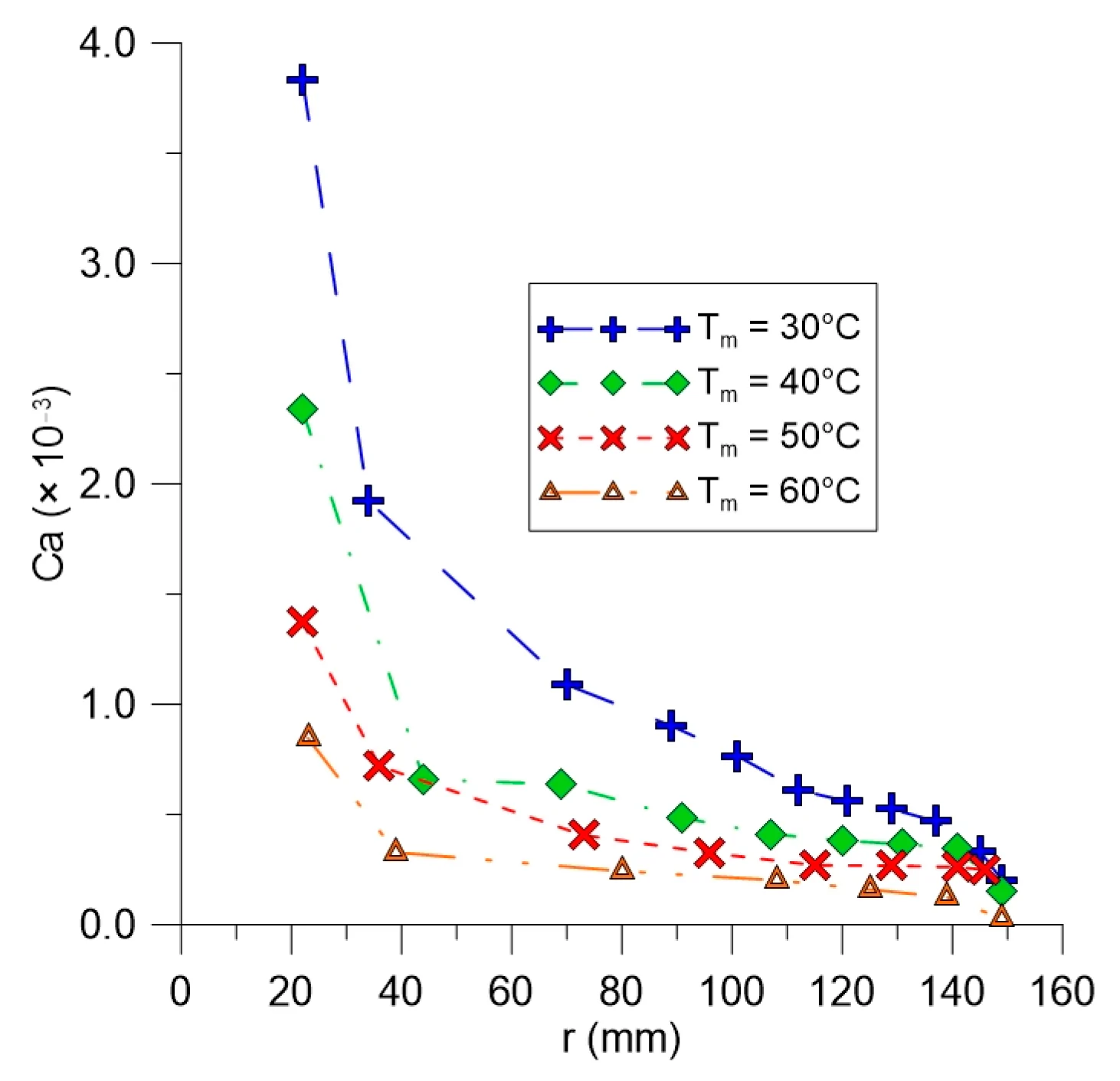
Capillary number versus radial position of the flow front, for injection under a constant pressure of 2.0 bar.

Initially, the operating condition of injection at constant gauge pressure equal to p_inj_ = 2.0 bar was simulated, with an injection temperature equal to 23 °C and a mold heating temperature between 30 and 60 °C, obtaining the curves shown in Figure 13, which relate the capillary number, *Ca*, to the radial position of the flow front, *r*.

It was found that the ideal capillary number range, between 0.5 × 10^−3^ and 1.7 × 10^−3^, where better filling of the voids in the fibrous reinforcement [26,27,28] is expected, was more present in the simulations where the mold heating temperature was lower, with the best results obtained where this temperature was 30 °C. It is important to emphasize that there is a complex balance of parameters for determining the capillary number, since this number depends on the dynamic viscosity of the resin at the flow front, which increases with time (due to curing effect) but decreases with increasing radial position (due to mold heating effect), and the superficial velocity of the flow front, which decreases with radial position and also decreases with increasing dynamic viscosity of the resin.

**Figure 14:**
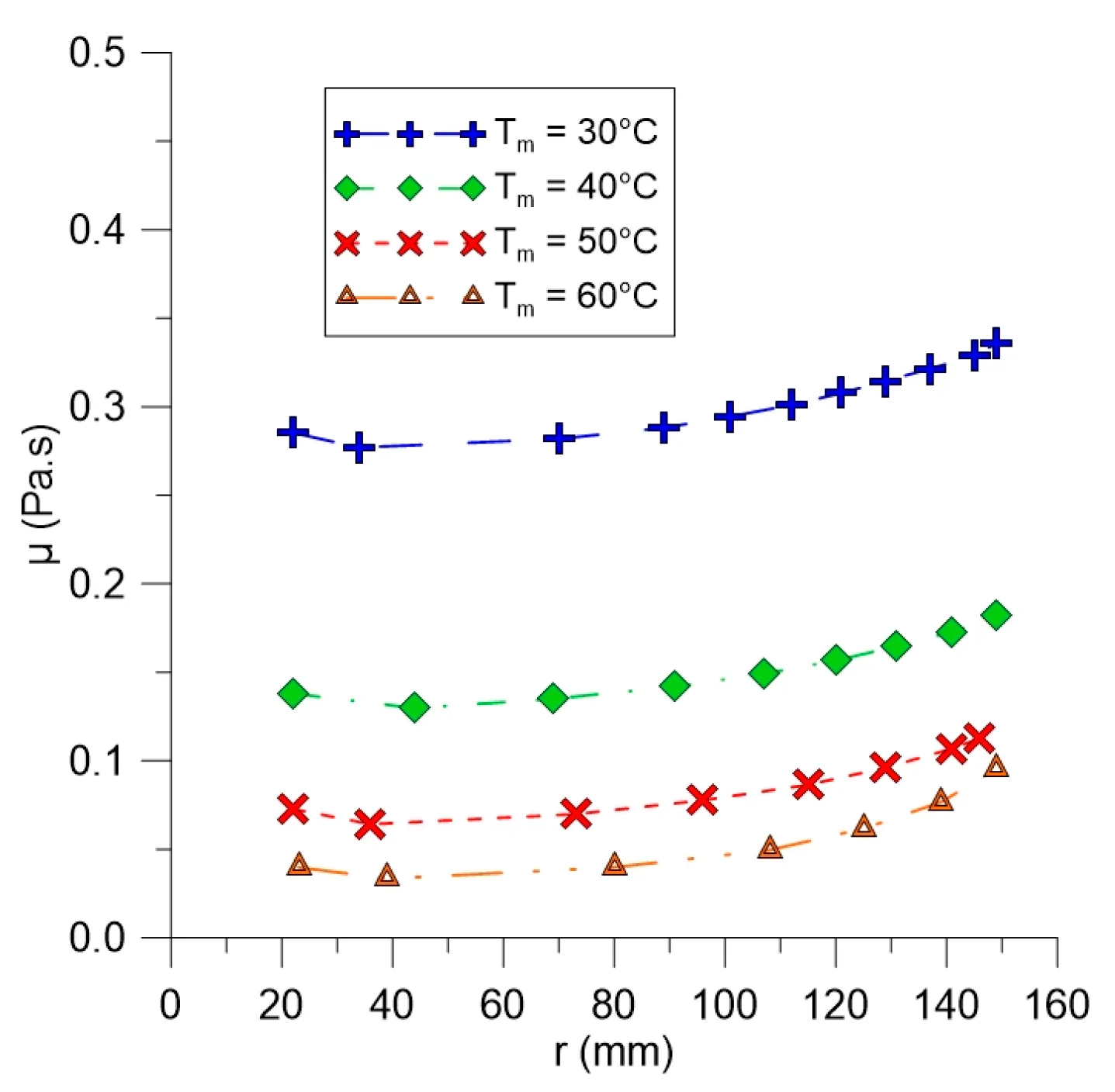
Dynamic viscosity of the resin at the flow front versus radial position, for injection under a constant pressure of 2.0 bar.

**Figure 15:**
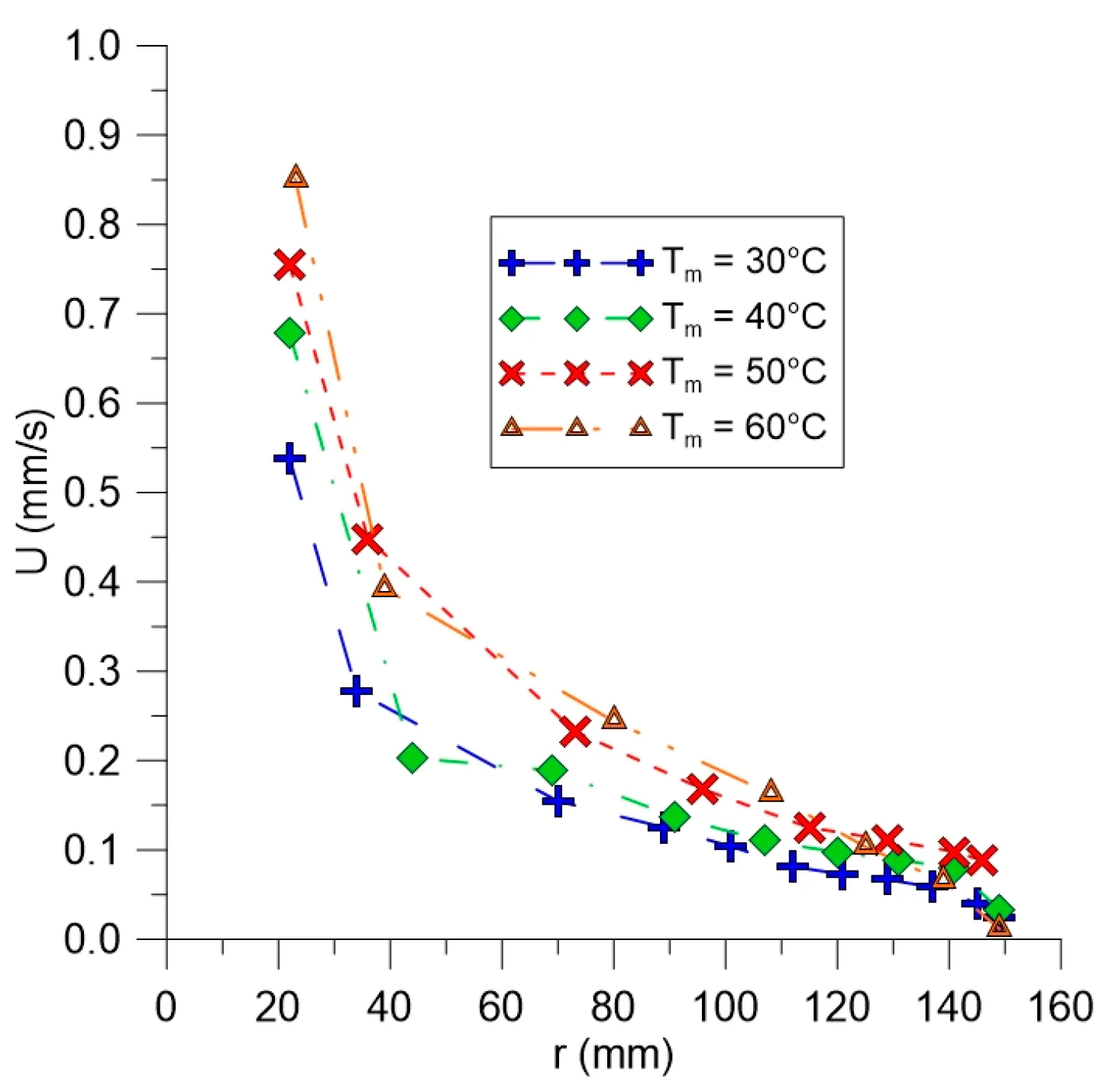
Superficial velocity of the flow front versus radial position, for injection under a constant pressure of 2.0 bar.

Since the capillary number is a function of the superficial velocity of the flow front, *U*, and the dynamic viscosity of the resin, µ, curves of these parameters were also plotted as a function of radial position for injection at a constant pressure of 2.0 bar. It can be observed that the capillary number is lower for higher mold heating temperatures due to the significant drop in the dynamic viscosity of the resin at the fluid front as this temperature increases (Figure 14). This capillary number also decreases with increasing radial position due to the drop in the superficial velocity of the flow front as the radius increases (see Figure 15). This trend aligns with previous findings by [23], which indicate that in production processes where there is constant injection pressure, the flow front velocity drops as the impregnated length advances, generating a capillary number field within the mold.

Based on the results shown in Figure 13, an adjustment equation for the capillary number versus mold temperature (°C) and radial position in the mold (mm) was developed for injection at a constant pressure of 2.0 bar and is shown below.

(20) $$Ca=\frac{0.131211-0.001973{\;T}_{m}}{r}$$

where R^2^ = 94.65%.

**Figure 16:**
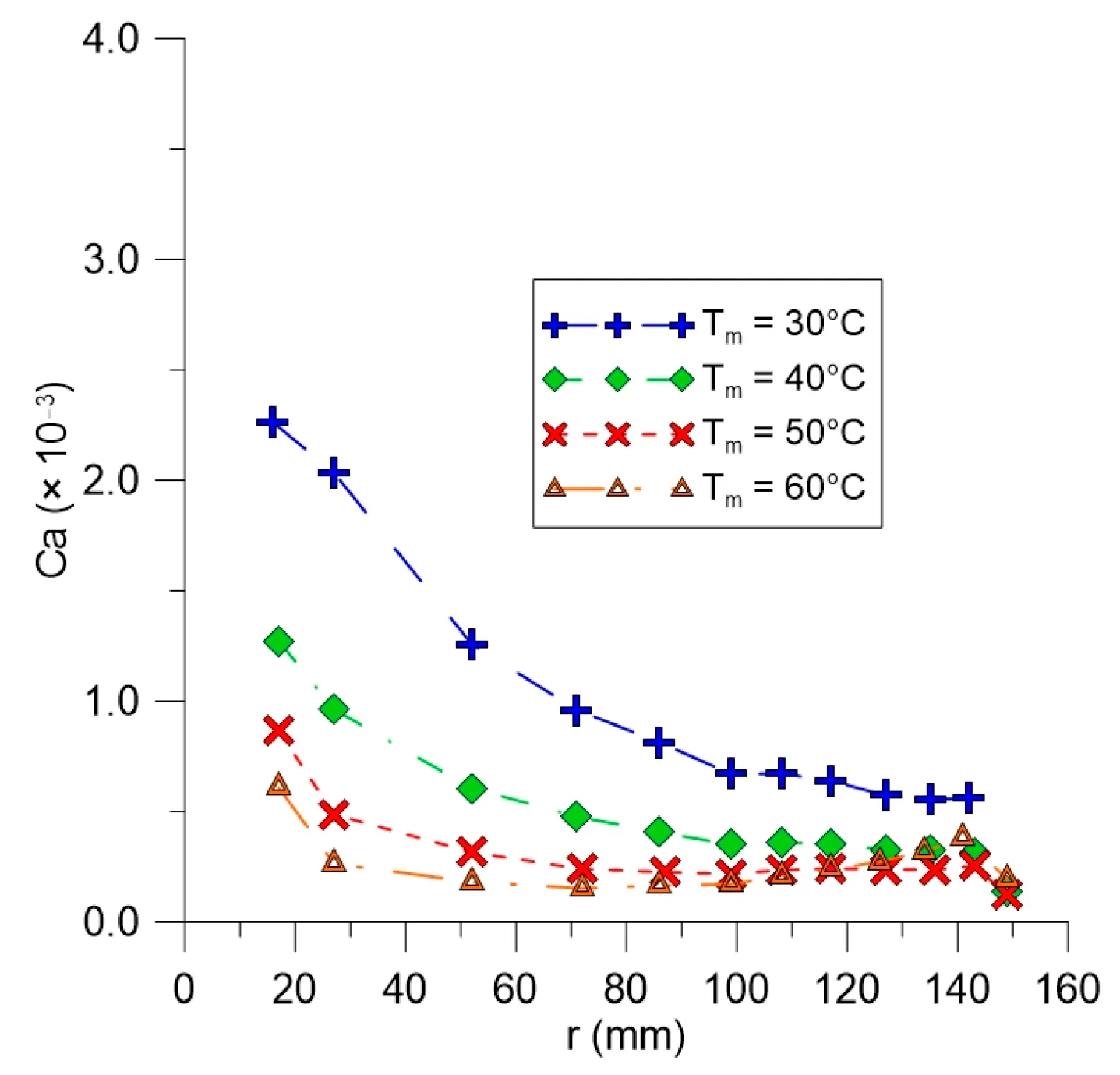
Capillary number versus radial position at the flow front, for injection under a constant volumetric flow rate of 3.5 × 10^−8^ m^3^/s.

Subsequently, the injection operating condition was simulated at a constant volumetric flow rate of Q_inj_ = 3.5 × 10^−8^ m^3^/s, with an injection temperature of 23 °C and a mold heating temperature ranging between 30 and 60 °C. From the simulations, the curves shown in Figure 16 were obtained, which relate the capillary number to the radial position of the flow front. By analyzing this figure, we can see similar behavior to that observed in the simulations with constant injection pressure, where the ideal capillary number range, between 0.5 × 10^−3^ and 1.7 × 10^−3^, was more present in the simulations where the mold heating temperature was lower, with better results being obtained, once again, when this temperature was 30 °C.

**Figure 17:**
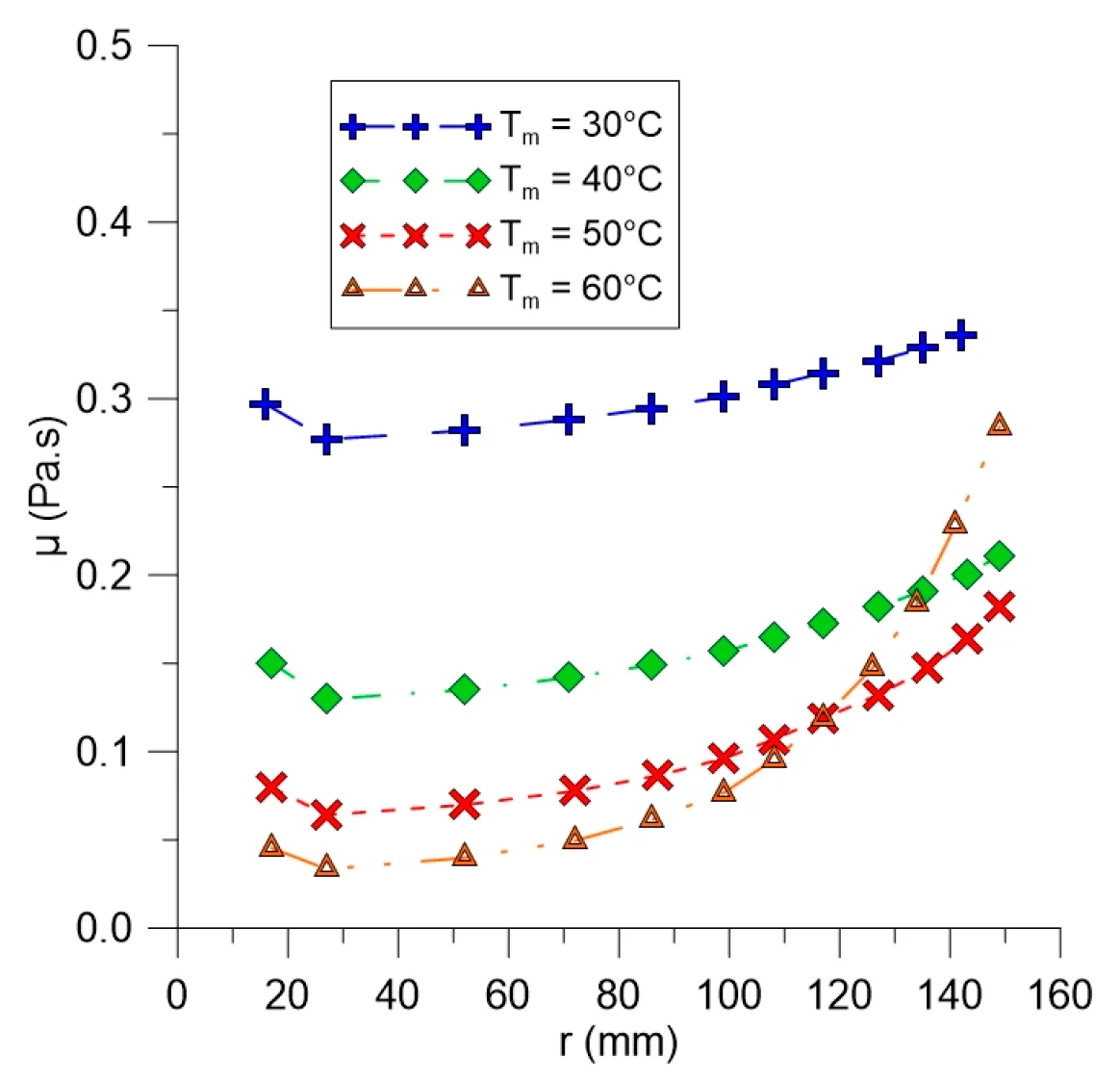
Dynamic viscosity of the resin at the flow front versus radial position, under a constant volumetric flow rate of 3.5 × 10^−8^ m^3^/s.

**Figure 18:**
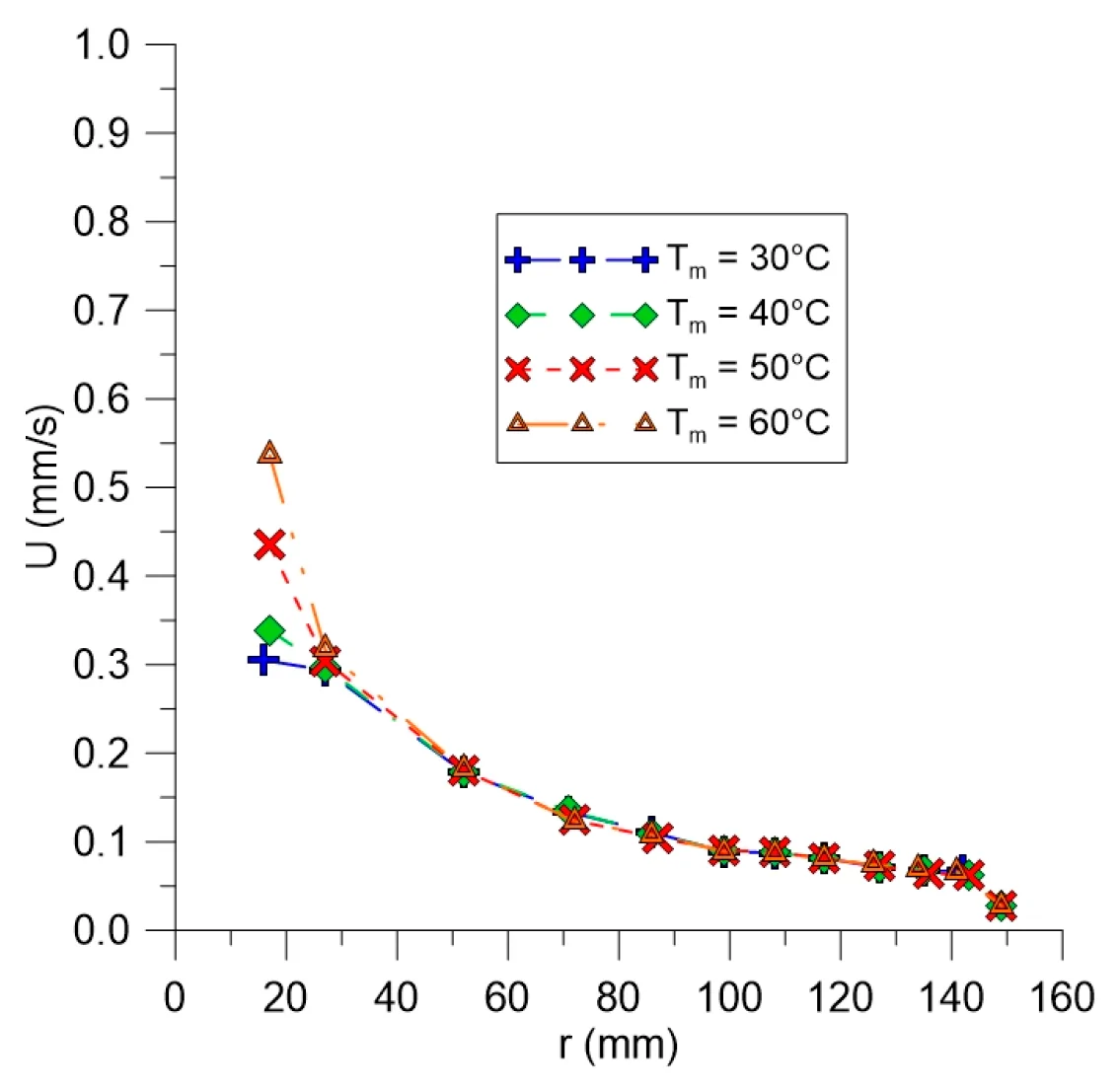
Superficial velocity of the flow front versus radial position, under a constant volumetric flow rate of 3.5 × 10^−8^ m^3^/s.

The superficial velocity of the flow front and the dynamic viscosity of the resin were also plotted versus radial position for the injection at a constant volumetric flow rate of 3.5 × 10^−8^ m^3^/s. As observed for the condition of resin injection at constant pressure, the capillary number is lower at higher mold heating temperatures due to the significant drop in the resin dynamic viscosity at the fluid front as this temperature increases (Figure 17), and this capillary number also decreases with increasing radial position due to the drop in the flow front superficial velocity as the radius increases (Figure 18). It should be noted that the viscosity behavior for a mold heating temperature of 60 °C, shown in Figure 17, is consistent with expectations, since the epoxy resin viscosity model used (Equation (14)) predicts greater increases in viscosity for longer process times, as the resin temperature increases.

From the analysis of Figure 13 and Figure 16, it also became evident that, in injection molding with a constant volumetric flow rate, compared to injection molding with constant pressure, lower capillary number levels were observed in smaller radii, with greater uniformity of the capillary number observed along the entire radius, for all mold heating temperatures. This is an indication that injection at constant volumetric flow would provide more uniform composites regarding the presence of voids along the radius.

Based on the results shown in Figure 16, an adjustment equation for the capillary number versus mold temperature (°C) and radial position within the mold (mm) was developed for injection at a constant volumetric flow rate of 3.5 × 10^−8^ m^3^/s, and is shown below.

(21) $$Ca=\frac{0.099785-0.001509{\;T}_{m}}{r}$$

where R^2^ = 94.28%.

**Figure 19:**
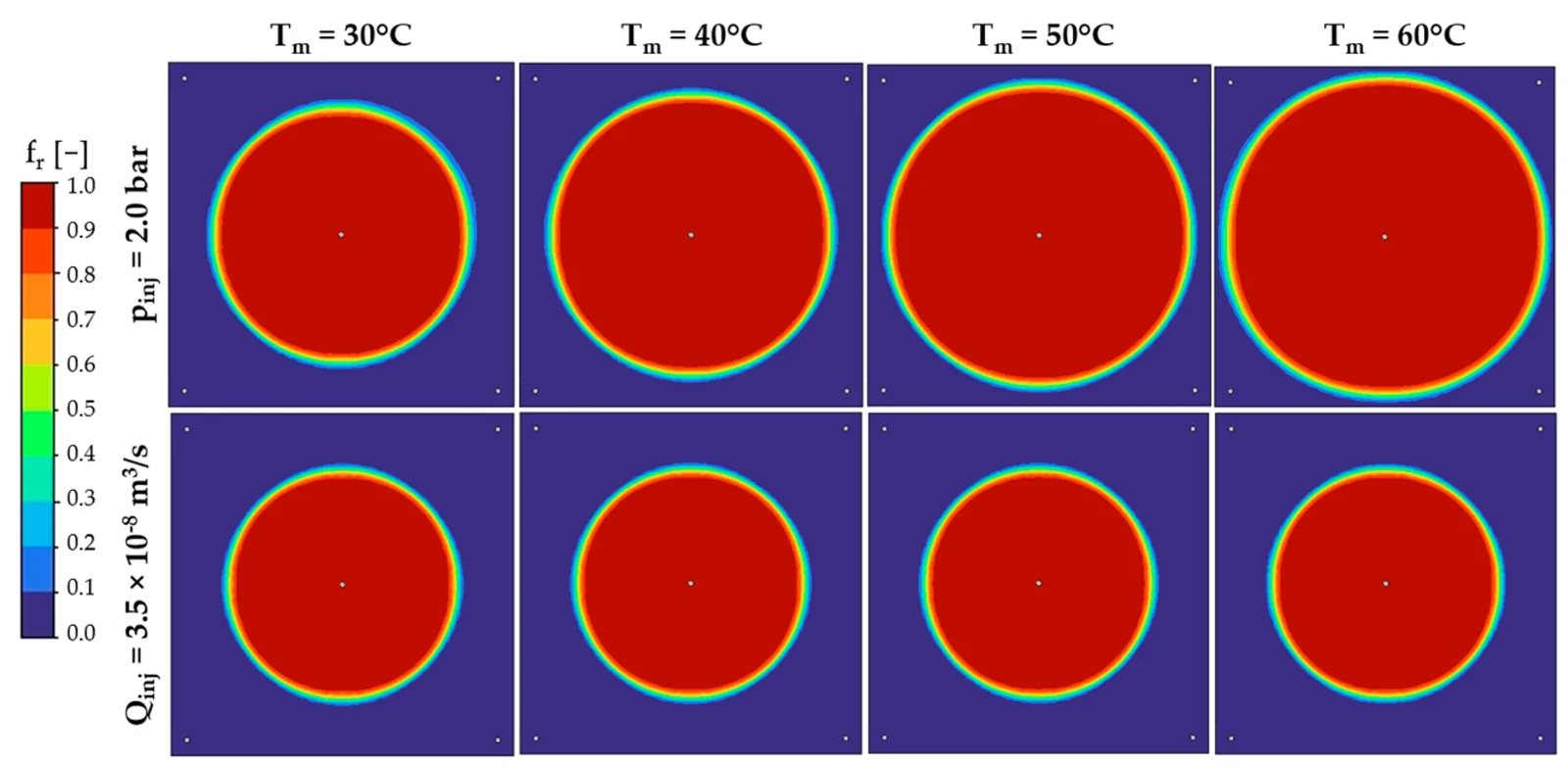
Volumetric fraction distribution of resin inside the mold at t = 500 s, for different mold temperatures and injection conditions.

Figure 19 shows the volumetric fraction distribution of resin inside the mold at t = 500 s, for different mold temperatures and injection conditions (gauge pressure or constant volumetric flow rate). Analyzing these images, a perfectly radial flow is observed until the injected fluid reaches the mold walls, since the fibrous medium is considered isotropic, homogeneous, and with constant porosity. If the fibrous medium exhibited non-constant porosity, the fluid flow front would likely develop a non-circular distribution, giving rise to fingering patterns. As the mold temperature increases, the flow front advances faster when injection occurs at constant pressure, and the flow front is approximately constant for injection at constant volumetric flow rate. This occurs due to the fact that in injection at a constant volumetric flow rate, as the mold temperature increases, there is greater mobility of the resin in the mold, which induces a reduction in the injection pressure to maintain the injection condition of constant volumetric flow rate. For comparison, the average injection pressure values throughout the entire process, from the beginning until the resin reaches the mold wall, were: 1.8 bar at T_m_ = 30 °C, 1.4 bar at T_m_ = 40 °C, 1.2 bar at T_m_ = 50 °C, and 1.1 bar at T_m_ = 60 °C. At 500 s, the injection pressure values were: 2.0 bar at T_m_ = 30 °C, 1.5 bar at T_m_ = 40 °C, 1.2 bar at T_m_ = 50 °C, and 1.1 bar at T_m_ = 60 °C.

**Figure 20:**
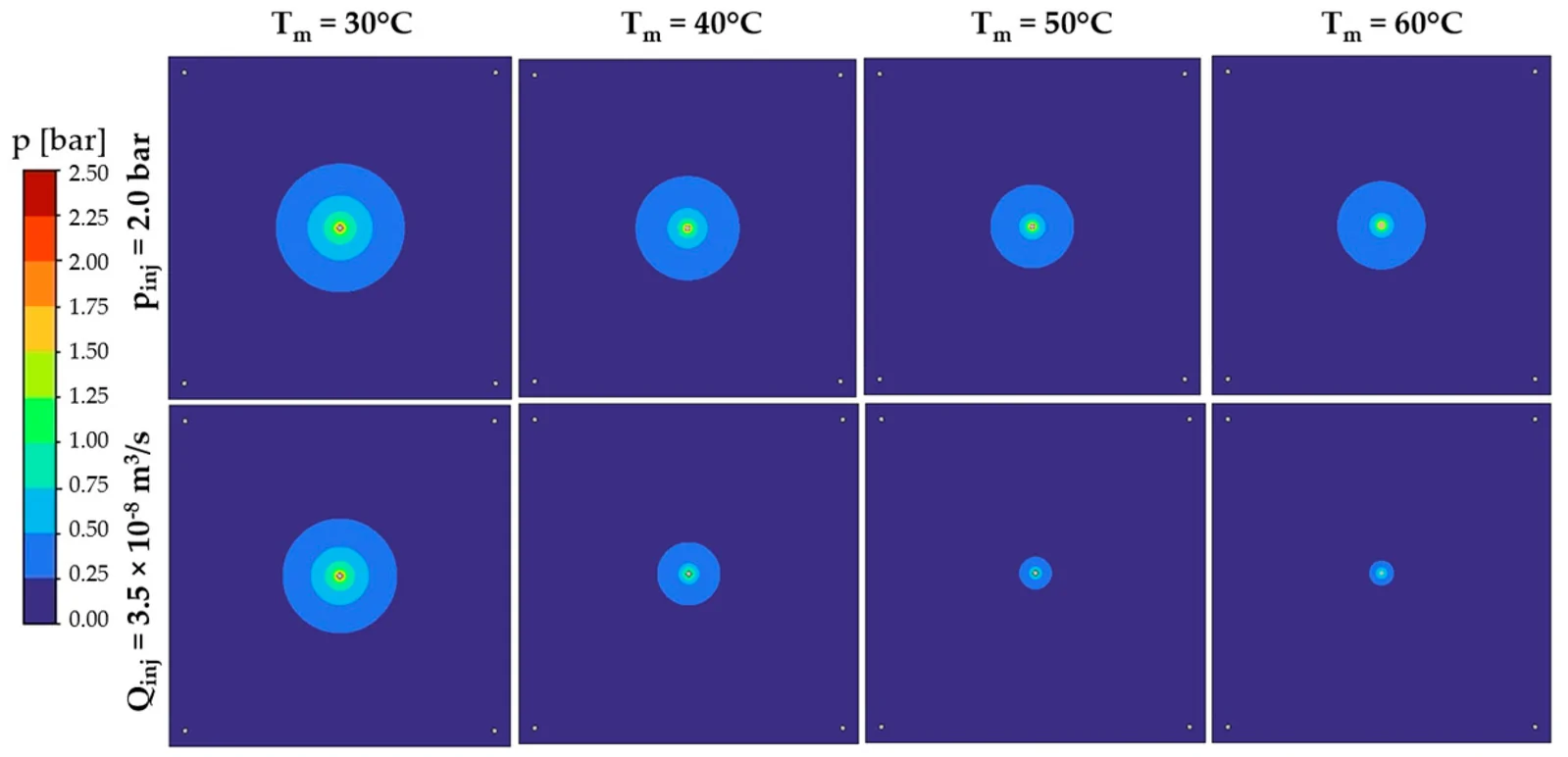
Gauge pressure distribution inside the mold at t = 500 s, for different mold temperatures and injection conditions.

Figure 20 shows the gauge pressure distribution in the mold at t = 500 s, for different mold temperatures and injection conditions (constant gauge pressure or constant volumetric flow rate). After analyzing these images, it can be verified that there is a maximum pressure at the injection gate along with a negative pressure gradient as the flow front radius increases. For injection at constant pressure, a more uniform pressure gradient is observed as the mold temperature increases.

**Figure 21:**
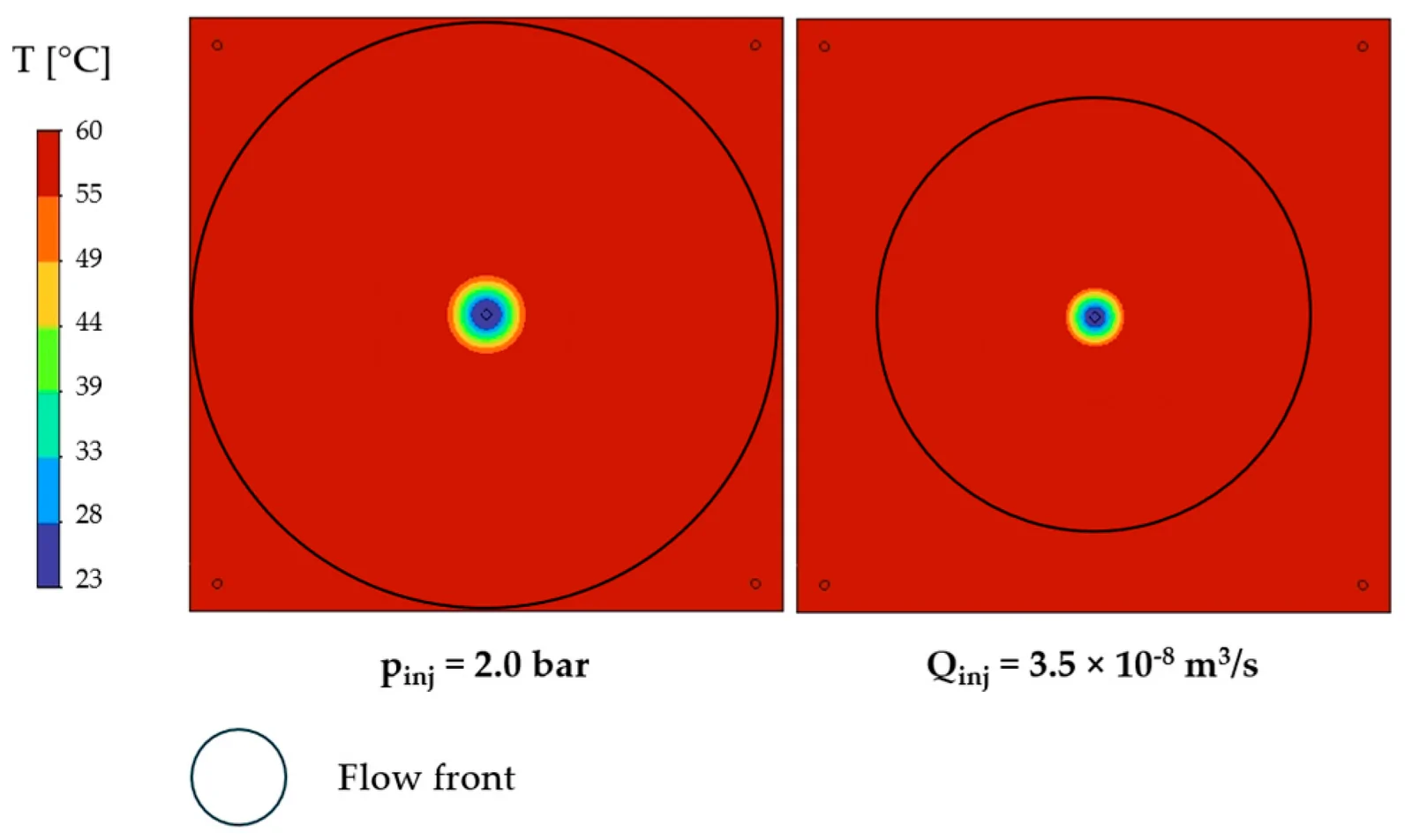
Temperature distribution of the resin inside the mold at T_m_ = 60 °C and t = 500 s, for different injection conditions.

Figure 21 illustrates the temperature distribution of the resin along the midline of the mold thickness (1 mm) at T_m_ = 60 °C and t = 500 s, for different injection conditions (gauge pressure or volumetric flow rate constant). Analyzing these images, very similar fields are verified, with quick heating of the resin, reaching the mold wall temperature (60 °C), because of the high contact area between the heated mold and the fluid flowing through the mold and the reduced thickness of the mold. The resin reaches the mold temperature (60 °C) at r = 20 mm for injection at constant pressure and at r = 15 mm for injection at constant volumetric flow rate.

### 3.3. Strengths and Limitations of the Model

The modeling proposed in this work exhibits robustness in its application to predict composite manufacturing using the RTM process. It accurately captures resin flow in a fibrous medium under multiphase, three-dimensional, and time-dependent non-isothermal conditions, including the sorption of resin within the micropores of the fibrous reinforcement. This model enables the evaluation of a wide variety of process parameters—from the porous medium characteristics to resin properties—providing deeper insight into the process. Such understanding is highly relevant for both practical applications and RTM experiments, supporting process optimization.

While the proposed model has several advantages, it is essential to point out its main limitations, which may be considered in future research efforts:

(a) Because of the use of a fixed sorption source term in the mathematical model, the saturation limit of the fiber micropores is not identified;
(b) The influence of resin infiltration into individual fibers, such as fiber swelling, is not considered;
(c) Various microscopic phenomena during fiber impregnation (e.g., trapped microvoids or void migration) were not considered;
(d) Fiber characteristics such as size, geometry, orientation, motion, packing arrangement, inter-fiber interactions, and preform deformation are not taken into account;
(e) Variations in mold thickness and deformation caused by internal pressure and temperature are disregarded, meaning mechanical and thermal effects on mold geometry are not considered;
(f) Exothermic heat of reaction generated during epoxy curing was omitted from the energy conservation equation, which may affect temperature fields during long injection cycles.

## 4. Conclusions

This work investigated the transient thermo-fluid behavior of resin flow during the Resin Transfer Molding (RTM) process, considering resin absorption by the reinforcement micropores through a CFD-based numerical framework. The study employed a non-isothermal, multiphase model with variable resin viscosity, including curing effects, to analyze the influence of mold heating temperature and injection strategy on mold filling and the capillary number field.

The results demonstrated that the proposed model successfully captured the combined effects of heat transfer, resin viscosity variation, multiphase flow, and resin absorption during mold filling. The analysis showed that injection under a constant volumetric flow rate (3.5 × 10^−8^ m^3^/s) produced a more uniform capillary number distribution along the flow path than injection under a constant gauge pressure (2.0 bar), indicating a lower variation in the potential for void formation throughout the mold.

The simulations also showed that increasing the mold heating temperature reduced resin viscosity and consequently shortened the mold filling time. However, lower mold temperatures promoted capillary numbers predominantly within the range of 0.5 × 10^−3^ to 1.7 × 10^−3^, which has been associated in the literature with a lower likelihood of void formation. Therefore, for the operating conditions investigated, the results indicate a trade-off between reducing manufacturing time and minimizing the potential occurrence of voids.

Overall, the study demonstrates how the combined analysis of the capillary number field and process operating conditions can support the selection of injection strategies and mold temperatures for RTM processing. These findings contribute to a better understanding of the physical mechanisms governing mold filling and provide useful information for process optimization.

Future work could focus on incorporating a resin absorption model that accounts for the flow direction relative to the fiber architecture, as well as capillary pressure effects between the resin and the entrapped air, in order to further improve the predictive capability of the numerical model.

## Figures and Tables

**Table 1:** Physical parameters of the porous medium used in numerical study.

Property	Value
Porosity (-) ^1^	0.6
Density (kg/m^3^) ^2^	2575
Permeability (m^2^) ^3^	5.02 × 10^−11^
Specific heat (kJ/kg·K) ^2^	0.8025
Thermal conductivity (W/m·K) ^2^	1.275

^1^ [54], ^2^ [55], ^3^ [50].

**Table 2:** Absorption source term of the porous medium used in simulations.

Mold Temperature (°C)	*s* (s^−1^) ^1^
30	0.57 × 10^−17^
40	0.13 × 10^−9^
50	0.24 × 10^−6^
60	0.84 × 10^−5^

^1^ Equation (3).

**Table 3:** Resin thermo-physical properties used in numerical simulations.

Property	Value
Dynamic viscosity (kg/m·s)	Equation (14)
Density (kg/m^3^) ^1^	1200
Specific heat (kJ/kg·K) ^2^	1.68
Thermal conductivity (W/m·K) ^3^	0.2
Surface tension (N/m) ^4^	0.04

^1^ Reference [56], ^2^ Reference [57], ^3^ Reference [58], ^4^ Reference [59].

**Table 4:** Air thermo-physical properties used in numerical simulations [60].

Property	Temperature (°C)			
	30	40	50	60
Dynamic viscosity (kg/m·s)	1.8680 × 10^−5^	1.9148 × 10^−5^	1.9608 × 10^−5^	2.0061 × 10^−5^
Density (kg/m^3^)	1.1649	1.1275	1.0925	1.0597
Specific heat (kJ/kg·K)	1.0065	1.0069	1.0074	1.0081
Thermal conductivity (W/m·K)	0.0263	0.0271	0.0278	0.0285

**Table 5:** Results obtained in V&V simulations.

Situation	Mold Condition	*s* (s^−1^)	*t_b_* (s)	*t_bv_* (s)	Deviation (%) ^1^
Verification	Unheated	1.0 × 10^−7^	561	570	1.60
Validation	Unheated	0.0 × 10^−4^	1000	1043	4.30
Validation	Heated (40 °C)	0.1 × 10^−4^	790	794	0.51
Validation	Heated (60 °C)	0.5 × 10^−4^	780	775	−0.64

^1^ [(*t_bv_ − t_b_*)/*t_b_*] × 100%.

## Data Availability

The original contributions presented in this study are included in the article. Further inquiries can be directed to the corresponding author.

## Abbreviations

The following abbreviations are used in this manuscript:

CFD	Computational Fluid Dynamic
GCI	Grid Convergence Index
RMS	Root Mean Square
RTM	Resin Transfer Molding
V&V	Verification & Validation
